# Comparative analysis of GAN-based augmentation pipelines for Parkinson's hypokinetic dysarthria severity detection using deep learning

**DOI:** 10.3389/frai.2026.1951063

**Published:** 2026-10-20

**Authors:** Aurobindo S, Prakash Ramachandran, Rajesh Kumar M

**Affiliations:** 1 School of Electronics Engineering, Vellore Institute of Technology, Vellore, India; 2 Department of Computer Engineering, VIT Mauritius, Pierrefonds, Mauritius

**Keywords:** class imbalance, convolutional neural network (CNN), dysarthria severity classification, GAN-generated image, GAN-generated speech, low-frequency cepstrogram

## Abstract

Class imbalance is a common problem in speech-based severity detection, which can result in biased models and poor recognition of minority severity classes. Generative Adversarial Networks (GANs) have been widely used for data augmentation, but very few studies have comparatively evaluated GAN-based augmentation at different stages of the speech-processing pipeline for assessing Parkinson's hypokinetic dysarthria severity. This paper compares GAN-generated speech (GGS) and GAN-generated image (GGI) augmentation methods for addressing class imbalance in low-frequency cepstrogram-based severity detection using the PC-GITA dataset. GAN-based augmentation was used to generate balanced training datasets using two different augmentation pipelines. The original speech and synthetic speech generated using GGS were converted into low-frequency cepstrogram representations, whereas GGI directly generated low-frequency cepstrogram images. These representations were used as inputs to a CNN trained and evaluated under Speaker-independent (SI) and Speaker-dependent (SD) conditions. In the speaker-independent evaluation, repeated five-fold speaker-stratified cross-validation with five independent random seeds yielded a mean accuracy of 56.47% ± 3.40% for GGS compared to 55.47% ± 3.35% for GGI and 53.13% ± 3.26% for the original-dataset baseline. GGS showed higher mean accuracy, precision, and specificity, whereas GGI showed higher mean recall and F1-score. In the speaker-dependent evaluation, GGI achieved the highest mean accuracy of 76.48% ± 4.09% compared to 73.04% ± 3.63% for GGS and 73.04% ± 3.25% for the original-dataset baseline. However, the speaker-dependent results represent an optimistic upper-bound evaluation because speaker identity was shared between the training and evaluation partitions. In general, the two GAN-based augmentation pipelines showed potential to address class imbalance under the conditions studied, while their relative performance depended on the evaluation protocol and the classification measure.

## 1 Introduction

Parkinson's disease (PD), a progressive neurodegenerative disorder affecting a significant percentage of the older adult population, results in progressive motor and non-motor disability including speech and language impairments (Munoz-Vigueras et al., 2021; Brabenec et al., 2017). Hypokinetic dysarthria is common in PD and is characterized by soft voice, monotone speech, imprecise consonants, hoarseness or breathiness, irregular pauses and degraded prosody, all of which reduce intelligibility and everyday communication (Moya-Galé and Levy, 2019; Orozco-Arroyave et al., 2014; Steurer et al., 2022). These speech changes are consistently reported as major determinants of quality of life in PD and have triggered the development of objective speech-based markers and automated assessment tools (Moya-Galé and Levy, 2019; Zhang, 2025). Recent studies have shown that acoustic analyses of PD speech can effectively discriminate patients from healthy controls (Munoz-Vigueras et al., 2021; Amato et al., 2021; Perrone et al., 2026) and that certain speech features are sensitive to disease severity and progression. Studies that use recordings of isolated words and sustained vowels have shown that the multi-level features extracted from full signals, vocalized portions and initial or final transitions in phonatory, articulatory and prosodic domains capture important manifestations of hypokinetic dysarthria and allow robust PD vs. healthy classification (Orozco-Arroyave et al., 2014; Amato et al., 2021; Perrone et al., 2026). Additional recent findings demonstrate that vowel space measures and prosodic descriptors can detect early, sub-clinical speech atypicality in PD speakers labeled as non-dysarthric, suggesting that acoustic markers may precede the formal clinical diagnosis of dysarthria (Roland et al., 2023; Nylén, 2025). A major challenge for automatic assessment is not only binary detection but also reliable multi-class severity grading. Acoustic severity is correlated with changes in brain areas involved in speech processing, and studies that characterize mild and moderate PD dysarthria severity report systematic differences in voice sound level, dysphonia, motor symptoms, and neural measures (Steurer et al., 2022; Zhang, 2025; Gómez et al., 2021). Also, automated acoustic models of dysprosody severity demonstrated that prosodic and cepstral features can approximate expert ratings, indicating that severity information is embedded in specific acoustic patterns that machine learning algorithms can learn (Nylén, 2025; Gómez et al., 2021). These results advocate for severity-oriented frameworks that link model outputs to clinically interpretable acoustic markers rather than straightforward disease and no-disease labels. At the same time, automatic speech recognition (ASR) and related artificial intelligence (AI) systems have been investigated as tools for measuring intelligibility and for remote monitoring in PD. Pilot studies have shown that cloud-based recognizers can discriminate dysarthric and healthy speech under realistic noise conditions, if appropriate prompts and noise controls are used (Goudarzi and Moya-Galé, 2021; Nudelman et al., 2025). However, these systems are still susceptible to data sparsity, noise and distributional shifts, especially for pathological speech, and their performance can differ substantially across speakers and severity levels (Goudarzi and Moya-Galé, 2021; Nudelman et al., 2025).

One of the main challenges in researching dysarthric speech is the lack of labeled data, especially for people with more severe forms of dysarthria (Steurer et al., 2022; Jin et al., 2023). To address this, researchers have begun using generative models such as Generative Adversarial Networks (GANs) and text-to-speech synthesis to create additional training data for speech recognition systems that help people with disordered or pathological speech (Jin et al., 2023; Chu et al., 2023; Leung et al., 2024; Bhat and Strik, 2025; Soleymanpour et al., 2024). Newer approaches, such as variational auto-encoder GAN frameworks, have worked better than older data-perturbation methods on standard dysarthria datasets. Other methods use encoder-decoder GANs to convert pathological speech patterns, as measured by mel-spectrograms, into more typical-sounding ones, thereby making the speech clearer and more understandable (Jin et al., 2023; Chu et al., 2023). Recently, combining text-to-dysarthric-speech synthesis with adversarial training has made it possible to generate extra dysarthric speech data, which helps reduce the gap in accuracy between speech recognition systems customized for a single speaker and those designed for a wider range of speakers (Leung et al., 2024; Bhat and Strik, 2025). There are a number of GAN-based augmentation studies that have focused on running or continuous speech (Jin et al., 2023; Chu et al., 2023), but sustained phonation has particular advantages for adversarial generative modeling of pathological speech. First, sustained vowels are quasi-stationary and phonetically invariant, leading to a relatively low-dimensional and structurally consistent target distribution. This reduces the burden on the generator to simultaneously capture linguistic content, coarticulation and phonatory pathology, and allows it to focus instead on laryngeal and phonatory irregularities, which are key to the analysis of pathological voice (Gerratt et al., 2016; Tsanas et al., 2021). Second, the fixed short duration of a sustained vowel task naturally suits the fixed-dimensional input and output tensors required by convolutional GAN architectures, whereas running speech of variable length often needs cropping, padding or alignment procedures that can complicate spectro-temporal modeling and destabilize adversarial training (Wali et al., 2022). Third, by isolating the phonatory subsystem from the articulatory and prosodic subsystems, the number of failure modes available to the GAN is reduced. A generator trained on continuous speech also has to preserve intelligible phonetic structure, rhythm, and prosody. This is a substantially harder generative problem under limited training data and is more prone to artifacts or mode collapse (Wali et al., 2022; Cobbinah et al., 2025). For these reasons, sustained vowel phonation is a well-justified substrate for GAN-based augmentation pipelines in pathological speech modeling.

Apart from waveform-based augmentation, the image-based approaches treat speech signals as time-frequency images and employ convolutional neural networks (CNNs) for pathological voice detection (Amato et al., 2021; Jegan and Jayagowri, 2024; Rehman et al., 2024). In such systems, spectrograms or cepstrograms are represented as 2D images encoding spectral and temporal variations, and CNNs are trained to learn discriminative spatial patterns related to voice disorders (Amato et al., 2021; Jegan and Jayagowri, 2024). According to reviews of AI techniques for voice pathology, these image-based architectures are useful for extracting the non-stationary features of pathological speech and for early detection of subtle voice impairment (Jegan and Jayagowri, 2024; Moulic et al., 2024; Aurobindo et al., 2025) performed a comparative study of different time-frequency image representations in three different forms such as standard, bifurcated standard and compact forms for detection and severity classification of dysarthria. The standard form images are spectrograms and cepstrograms. The bifurcated standard form images are divided into two parts: one part contains the low frequency and the other contains the high frequency, giving low-frequency spectrograms and cepstrograms and high-frequency spectrograms and cepstrograms. The compact form images are a cochleagram and a melscalogram. According to this study, the low-frequency cepstrogram outperformed all other metrics in detecting and classifying the severity of dysarthria. Thus, the main question of this study is methodological rather than purely clinical: given a fixed time-frequency representation and a fixed CNN architecture, how do two practical GAN-based class-balancing augmentation pipelines compare when augmentation is performed in the speech domain by generating synthetic pathological waveforms that are subsequently transformed into low-frequency cepstrogram images, or in the image domain by directly generating synthetic low-frequency cepstrogram images from the real low-frequency cepstrograms of the minority class? This question is evaluated by comparing the performance of the two GAN-based augmentation pipelines under the same minority-class balancing target, downstream CNN architecture, and evaluation protocols, while allowing each GAN pipeline to use its respective formulation and training configuration. However, the two GAN pipelines were not designed as a strictly controlled domain-only comparison. The speech representation was 128 × 640, whereas the image representation was 128 × 128, resulting in five times more representation elements in the speech-domain input. In addition, WGAN-GP requires gradient-penalty computation and additional critic optimization, which increases the computational requirements of adversarial training (Gulrajani et al., 2017). Recent comparative work on medical spectrogram generation has also reported higher GPU memory requirements for WGAN-GP than DCGAN under the same experimental setting (Barbosa-Casanova et al., 2026). Therefore, a computationally lighter GAN configuration was used for the speech domain. Hence, differences between GGS and GGI may reflect both the augmentation domain and the respective GAN formulation and training configuration. The comparison is therefore intended to evaluate the practical effectiveness of the two complete augmentation pipelines under their respective configurations rather than to isolate the effect of augmentation domain alone. Therefore, in this work, the actual speech and GAN-generated speech are transformed to low-frequency cepstrogram images, and for the GAN-generated image, the low-frequency cepstrogram of the minority class of the original speech was used to generate the synthetic images, which were used for the comparison of severity detection of hypokinetic dysarthria in PD using sustained vowels of PC-GITA dataset using three experimental conditions, namely (i) the actual low-severity and high-severity speech recordings, (ii) the speech generated using GAN to balance the under-represented dysarthria severity classes, and (iii) the images generated using GAN from the time-frequency representation highlight long-term spectral and vocal-source characteristics. This work systematically compares the support of these three modalities in automatic grading of hypokinetic dysarthria severity in speaker-dependent and speaker-independent configurations. This is based on dataset-based PD speech analysis (Orozco-Arroyave et al., 2014; Amato et al., 2021; Perrone et al., 2026; Gómez et al., 2021), the demonstrated utility of GAN-based augmentation and synthesis in disordered and pathological speech (Jin et al., 2023; Chu et al., 2023; Leung et al., 2024; Bhat and Strik, 2025; Soleymanpour et al., 2024), and the discriminative power of time-frequency image representations processed by CNNs (Amato et al., 2021; Jegan and Jayagowri, 2024; Rehman et al., 2024). In this work, GGI achieved a higher observed test accuracy of 61.67% compared with 59.17% for GGS, together with higher observed precision, recall, specificity, and F1-score in the speaker-independent evaluation. In the repeated speaker-independent evaluations, GGS obtained a higher mean accuracy than GGI, although variability was observed across folds and random seeds. GGS also obtained higher mean precision and specificity, whereas GGI achieved higher mean recall and F1-score. In speaker-dependent evaluation, GGI obtained a test accuracy of 73.13%, compared with 66.42% for the original dataset and 63.43% for GGS. Under speaker-dependent evaluation, GGI showed higher observed precision, recall, specificity, and F1-score than GGS. The repeated speaker-dependent evaluations further showed a higher mean accuracy for GGI than GGS and the corresponding original-dataset baseline, although variability was observed across the repeated evaluations. GGI also achieved higher mean recall, specificity, and F1-score than GGS, whereas GGS achieved higher mean precision. In the speaker-dependent evaluation, the paired comparison between GGI and the original dataset was statistically significant, whereas the corresponding comparison for GGS was not statistically significant. However, since speaker identity was shared between training and evaluation partitions in the SD protocol, these results are more of an optimistic upper bound rather than a measure of generalizable severity classification. The speaker-independent results, in which speakers are not shared between the training and evaluation partitions, are a more appropriate basis for assessing generalization. The results show that the relative performance of GGI and GGS depended on the evaluation protocol and classification measure within the experimental setting evaluated. The absolute classification accuracies of PD severity detection from isolated sustained vowels are generally lower than those reported for continuous or read speech tasks in the wider dysarthria severity literature where multi-class severity classification systems tested on the UA-Speech and TORGO datasets have achieved accuracies above 95% in speaker-dependent or utterance-level evaluation protocols (Javanmardi et al., 2024b; Varma et al., 2025; Remya et al., 2025). This difference may be related to the task design, including the use of sustained vowels, the binary severity definition, and the characteristics of the dataset, as discussed in Section 4.4. The key highlights of the study are summarized below:

- GAN-based augmentation pipelines are investigated to address class imbalance in low-frequency cepstrogram-based hypokinetic dysarthria severity classification.
- GAN-generated speech (GGS) and GAN-generated image (GGI) are compared as two distinct approaches for augmenting pathological speech data.
- The study explores augmentation at different levels of the processing pipeline, where GGS generates speech that is subsequently converted into low-frequency cepstrograms, whereas GGI directly generates low-frequency cepstrogram images.
- Both augmentation approaches are evaluated under speaker-independent and speaker-dependent classification protocols.
- The comparative results indicate that the relative performance of GGS and GGI varies with the evaluation protocol and classification measure, with GGI showing more favorable overall performance under the speaker-dependent protocol and GGS showing advantages in accuracy, precision and specificity in the repeated speaker-independent evaluation.

The structure of this paper is defined as follows: Section 2 provides related works, the methods and data of the work are provided in Section 3, the significant research findings are presented in Section 4, and the overall conclusions of the research are given in Section 5.

## 2 Related works

Acoustic analyses of PD speech, performed across various dataset, have described the typical characteristics of hypokinetic dysarthria and examined their usefulness for diagnosis and prognosis (Munoz-Vigueras et al., 2021; Orozco-Arroyave et al., 2014; Amato et al., 2021; Gómez et al., 2021). Reviews of dysarthria in PD highlight the high prevalence, a typical pattern of reduced loudness, monotone prosody, restricted fundamental-frequency range, consonant and vowel imprecision, breathiness and irregular pauses and the negative effects on intelligibility and social participation (Moya-Galé and Levy, 2019; Orozco-Arroyave et al., 2014; Zhang, 2025). Corpus-based analyses of isolated words, sustained vowels, and diadochokinetic sequences have identified sets of acoustic features in the areas of phonatory stability, pitch variability, rhythm, and articulation that effectively discriminate PD speakers from healthy controls, and that reflect underlying neuromotor dysfunction (Munoz-Vigueras et al., 2021; Brabenec et al., 2017; Amato et al., 2021; Perrone et al., 2026). More recent work has been on early and subtle speech biomarkers, and explicit severity grading Studies of vowel production have demonstrated that measures such as triangular vowel space area, vowel articulation index and PHI index distinguish PD speakers with dysarthria from healthy controls and that PHI shows reduced vowel-space organization and increased intra-category variability even in PD speakers classified as non-dysarthric, supporting early detection of subclinical impairment (Roland et al., 2023; Nylén, 2025). Mild and moderate dysarthria have been characterized and found to differ between severity groups in terms of voice sound level, dysphonia, motor scores, and neural measures (Steurer et al., 2022; Zhang, 2025; Gómez et al., 2021). Dysarthria severity has been found to correlate with resting-state functional activity and gray-matter volume in regions involved in auditory and speech processing (Steurer et al., 2022; Zhang, 2025; Gómez et al., 2021). Moreover, automated acoustic models for dysprosody severity based on prosodic features and Mel-frequency cepstral coefficients have also demonstrated that some prosodic and cepstral predictors can approximate expert ratings, suggesting that severity information can be captured by machine-learning models trained on carefully selected acoustic features (Nylén, 2025; Gómez et al., 2021). Parallel work has explored the feasibility of ASR and related artificial intelligence systems in terms of intelligibility measurement and remote monitoring in PD. Pilot studies of dysarthric vs. healthy speech in multi-talker babble noise have shown that cloud-based recognizers can measure speech intelligibility and detect differences between groups when noise conditions and linguistic prompts are controlled, but they also highlight significant sensitivity to noise and data distribution, especially for more severely impaired speakers (Goudarzi and Moya-Galé, 2021; Nudelman et al., 2025). These results emphasize the need for robust models that can accommodate inter speaker variability, noise, and severity imbalance in clinical or home deployments. To address the issues of data scarcity and data imbalance, several studies have proposed GAN-based and synthesis-based methods for disordered and pathological speech. Variational auto-encoder GAN frameworks for personalized dysarthric speech augmentation learn independent latent spaces for dysarthric features and phoneme context and have been shown to outperform speed perturbation and non-VAE GAN baselines on dysarthria dataset when training hybrid and end-to-end recognizers (Jin et al., 2023; Leung et al., 2024). Encoder-decoder GANs have enhanced intelligibility and content similarity on pathological voice database while preserving speaker identity (Chu et al., 2023) by converting pathological to normal mel-spectrograms and synthesizing personalized normal speech with neural vocoders. Two-stage augmentation pipelines are proposed combining GAN-based spectral modification, dysarthria-specific SpecAugment, and text-to-dysarthric-speech synthesis, to improve speaker-independent performance while allowing further fine-tuning in speaker-dependent or severity-dependent setups (Leung et al., 2024; Bhat and Strik, 2025). These approaches show that adversarial generative models and dysarthric speech synthesis can be used to successfully increase the amount and diversity of pathological training data, and improve the robustness of downstream ASR systems. But they are mostly evaluated based on recognition accuracy or conversion quality and very rarely by explicit severity grading of motor speech disorders. In contrast, severity-oriented acoustic studies in PD have emphasized the significance of vowel-space organization, prosodic variability, and cepstral features as clinically meaningful markers, suggesting that generative augmentation should be investigated not only for its effect on recognition, but also for its effect on these severity markers (Steurer et al., 2022; Roland et al., 2023; Nylén, 2025; Gómez et al., 2021). This direction is followed in the present work by mainly using GANs for balancing dysarthria severity classes and by analyzing severity detection performance in terms of acoustic markers and expert labels. Image-based approaches convert time-frequency representations of speech such as spectrograms or cepstrograms to two-dimensional images and use CNNs to detect voice pathology (Amato et al., 2021; Jegan and Jayagowri, 2024; Rehman et al., 2024). Studies that used mel-spectrogram images and optimized CNN architectures on multiple public voice pathology datasets reported high accuracy, showing stable, data-independent discriminative capability (Amato et al., 2021; Jegan and Jayagowri, 2024). Wider reviews of AI approaches in voice disorders emphasize that image-based models are able to capture subtle changes in spectral energy distributions, formant trajectories, and noise content associated with pathological voice production and can be used as non-invasive screening tools in clinical practice (Jegan and Jayagowri, 2024; Moulic et al., 2024). Studies of neuromotor-to-acoustic projection and multimodal data of speech, electromyography, and articulatory motion have demonstrated that jaw-tongue kinematics and formant dynamics are closely linked to hypokinetic dysarthria and that neuromotor deficits are richly reflected in the low-frequency and prosodic domains in PD (Brabenec et al., 2017; Zhang, 2025; Gómez et al., 2021). These results motivate image encodings that emphasize low-frequency cepstral information and prosodic structure and the use of CNNs to learn spatial patterns associated with dysarthria severity. However, existing systems are mostly image based and work on real spectrograms, not explicitly exploring how the images generated by GANs from speech descriptors compare to real images for severity detection, or how they interact with GANs generated audio in multimodal frameworks. In this work, these gaps are bridged by synthesizing images from low-frequency cepstrograms using GANs, training image-based models on these synthetic images, and comparing severity detection performance on real and GAN-generated speech in the same corpus. And contextualize this comparison within the broader literature on acoustic severity markers, generative augmentation, and image-based pathology detection (Munoz-Vigueras et al., 2021; Brabenec et al., 2017; Moya-Galé and Levy, 2019; Orozco-Arroyave et al., 2014; Steurer et al., 2022; Zhang, 2025; Amato et al., 2021; Roland et al., 2023; Nylén, 2025; Gómez et al., 2021; Goudarzi and Moya-Galé, 2021; Nudelman et al., 2025; Jin et al., 2023; Chu et al., 2023; Leung et al., 2024; Bhat and Strik, 2025; Soleymanpour et al., 2024; Jegan and Jayagowri, 2024; Rehman et al., 2024; Moulic et al., 2024) to offer a modality-comparative perspective on hypokinetic dysarthria severity detection that is in line with current trends in speech-based assessment and AI-assisted clinical decision-making. Recent studies have continued to investigate speaker-independent dysarthria severity classification and data augmentation. The study (Kim et al., 2025) investigated speech-synthesis-based augmentation for speaker-independent dysarthria severity classification using zero-shot text-to-speech and voice-conversion models, with the aim of improving classification when speaker-specific information is unavailable. Researchers (Suresh et al., 2025) investigated dysarthria severity classification with deep learning models using MFCC features. They proposed data augmentation strategy based on multi-voice synthesis and evaluated the approach under both speaker-dependent and speaker-independent settings. In the study, the authors (Kadirvelu et al., 2025) proposed a speaker-independent severity classification framework based on wav2vec 2.0 and a multi-task learning strategy designed to reduce reliance on speaker-specific characteristics. Their evaluation on the UA-Speech dataset used leave-one-subject-out cross-validation. More recent work authors (Alqam et al., 2025) investigated speech-disorder severity classification in patients with Parkinson's disease using acoustic features and machine-learning classifiers, with segment-level predictions aggregated to obtain subject-level classifications. These studies demonstrate continued interest in synthetic augmentation, speaker-independent evaluation, and automated severity assessment. Thus, comparison of GAN-based augmentation at different stages of the processing pipeline which are through synthesized speech and directly generated low-frequency cepstrogram representations is still less explored for Parkinson's disease hypokinetic dysarthria severity classification.

## 3 Methods and data

### 3.1 Dataset

The PC GITA (Orozco-Arroyave et al., 2014) is a Spanish speech corpus used for detecting the severity of hypokinetic dysarthria in Parkinson's speech. It consists of neurophysiological recordings from 50 PD patients and 50 healthy controls, matched for age and gender. The clinical assessments were obtained from UPDRS ratings provided by neurologist experts. PD speakers had disease durations ranging from 0 to 43 years, with a mean of 11 ± 10 years, and all recordings were made in the ON state, that is, within 3 h after morning medication. The corpus includes five speaking tasks: sustained vowels, isolated sentences, diadochokinetic (DDK) utterances, text reading, and monolog speech. The vowel task consisted of five vowels /a,e,i,o,u/ repeated three times per speaker. The sentence task consisted of six sentences. The DDK task consisted of quick repetition of syllables such as pa-ta-ka and related sequences. The read-text task consisted of a fixed dialogue between a doctor and a patient. The monolog task asked speakers to talk about everyday topics. In this study, only the vowel task was used for severity detection. The original sampling frequency was 44.1 kHz, and then downsampled for analysis to 8 kHz. For severity detection, only the PD speakers from the PC GITA corpus were used and healthy controls were not included as the task was to distinguish low vs. high dysarthria severity in patients. The PD speakers were divided into two severity classes, low and high, as listed in Table 1, based on the UPDRS speech score (UPDRS-S) provided in the PC-GITA clinical metadata. The low-severity class was defined as a speech score of 0–1, corresponding to normal or mildly affected speech, whereas the high-severity class was defined as a speech score of 2–3, corresponding to moderately or severely affected speech. The UPDRS-S ratings were obtained from the clinical metadata of the original PC-GITA corpus, and inter-rater reliability information was not available in the metadata used in this secondary analysis. Scores of 1 and 2 were retained according to the predefined severity threshold, with scores of 0–1 assigned to the low-severity class and scores of 2–3 assigned to the high-severity class; no additional borderline PD speakers were excluded. The model was evaluated under both speaker-independent and speaker-dependent protocols.

**Table 1:** Speaker distribution based on the classes.

	High severity	Low severity
Speaker ID	AVPEPUDEA00: 03, 10, 11, 14, 24, 25, 30, 31, 35, 37, 39, 41, 43, 46, 48, 51, 53, 55, 57, 59	AVPEPUDEA00: 01, 02, 05, 06, 07, 08, 09, 13, 15, 16, 17, 20, 21, 22, 23, 26, 27, 29, 32, 34, 38, 42, 45, 47, 49, 50, 52, 54, 56, 58

### 3.2 Proposed GAN for speech generation

The proposed framework uses a deep convolutional Generative Adversarial Network (GAN) to generate synthetic pathological sustained vowel speech signals of each vowel individually trained on log-magnitude spectrogram representations extracted from the PC-GITA dataset. GAN-based speech synthesis and spectrogram generation have shown to be effective for learning realistic acoustic structure from limited data and spectrogram-based adversarial generation has also been used successfully in speech synthesis and dysarthric speech modeling. The overall workflow, shown in Figure 1, included: (i) audio preprocessing and spectral representation, (ii) frequency-wise normalization, (iii) adversarial training using generator and discriminator networks, and (iv) spectrogram inversion to reconstruct the waveform.

**Figure 1:**
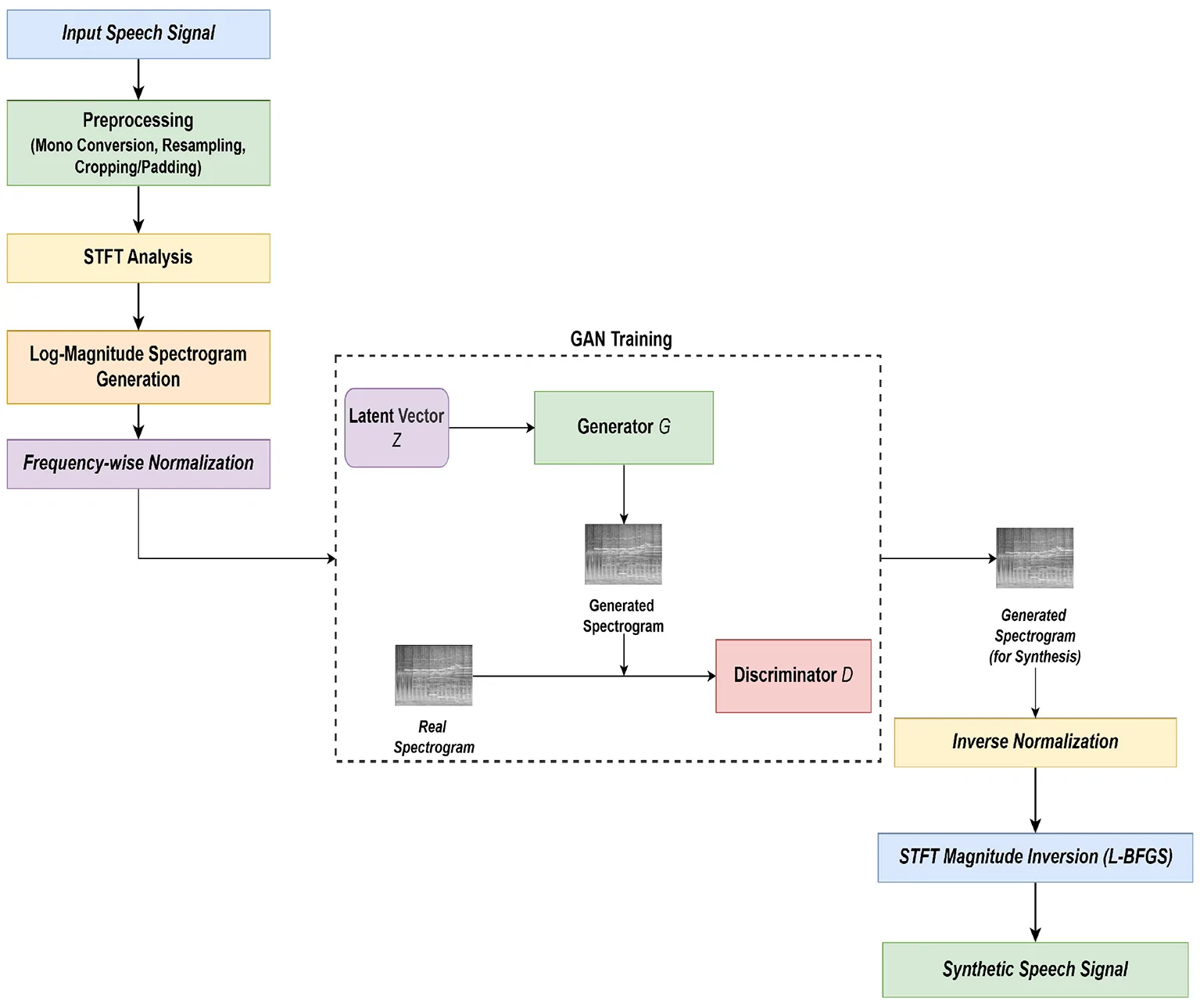
Overview of the proposed GAN-based pathological speech synthesis framework consisting of spectrogram extraction, adversarial learning, and waveform reconstruction.

#### 3.2.1 Audio Preprocessing and spectral representation

Let *x*(*n*) be a sustained vowel recording input. All recordings were initially converted to single channel and resampled to 8 kHz for uniformity throughout the dataset, a typical choice in speech-based Parkinson's disease studies and related speech synthesis pipelines. Amplitude normalization was then performed accordingly, as shown in Equation 1


$$\begin{array}{c} x_{n}\left(n\right)=\frac{x\left(n\right)}{\max_{k}|x\left(k\right)|+\epsilon } \end{array}$$ (1)

where ϵ = 10^−8^ was introduced to avoid division by zero.

All recordings were equalized to a fixed length of 640 STFT frames because the GAN architecture requires fixed dimensional inputs. Signals shorter than the required length were symmetrically zero-padded and longer signals were center-cropped. For time-frequency analysis the Short-Time Fourier Transform (STFT) was employed with a Hann window of length 256 samples, overlap of 128 samples and FFT size of 256 samples and the overall spectrogram extraction parameters is listed in Table 2. STFT representation is defined in Equation 2 as follows


$$\begin{array}{c} X\left(k,m\right)=\overset{N-1}{\underset{n=0}{\sum }}x\left(n\right)w\left(n-mH\right)e^{-j2\pi kn/N} \end{array}$$ (2)

where *w*(*n*) is the analysis window, *H* is the hop size, *k* is the frequency index, and *m* is the frame index. The magnitude spectrogram was computed using Equation 3


$$\begin{array}{c} S\left(k,m\right)=|X\left(k,m\right)| \end{array}$$ (3)

and logarithmic compression was applied to reduce spectral dynamic range as shown in Equation 4


$$\begin{array}{c} S_{\log}\left(k,m\right)=\log\left(S\left(k,m\right)+10^{-6}\right) \end{array}$$ (4)

The Nyquist frequency component was removed to obtain spectrogram images of dimensions 128 × 640, which served as inputs to the GAN model.

**Table 2:** Spectrogram extraction parameters.

Parameter	Value
Sampling frequency	8 kHz
Window type	Hann
Window length	256
FFT length	256
Overlap length	128
Hop length	128
Number of frames	640
Frequency bins	128
Spectral representation	Log-magnitude STFT

#### 3.2.2 Frequency-wise spectral normalization

The normalization was performed frequency-wise to maintain pathological spectral characteristics while improving numerical stability during training. For each frequency band, the mean and standard deviation were estimated across the whole training dataset using Equations 5, 6


$$\begin{array}{c} \mu_{k}=\frac{1}{N}\overset{N}{\underset{i=1}{\sum }}S_{log}^{\left(i\right)}\left(k\right) \end{array}$$ (5)

$$\begin{array}{c} \sigma_{k}=\sqrt{\frac{1}{N}\overset{N}{\underset{i=1}{\sum }}{\left(S_{log}^{\left(i\right)}\left(k\right)-\mu_{k}\right)}^{2}}+10^{-6} \end{array}$$ (6)

where *N* denotes the number of training samples. The normalized spectrogram was computed using Equation 7


$$\begin{array}{c} \hat{S}\left(k,m\right)=\frac{S_{log}\left(k,m\right)-\mu_{k}}{\sigma_{k}}. \end{array}$$ (7)

To match the output range of the generator, the normalized values were further scaled and clipped to the interval −1, 1 using Equation 8


$$\begin{array}{c} S_{\text{norm}}\left(k,m\right)=\max\left(\min\left(\frac{\hat{S}\left(k,m\right)}{3},1\right),-1\right) \end{array}$$ (8)

128 × 640 × 1 were the resulting input tensor dimensions.

#### 3.2.3 Generator architecture for speech

The generator network transformed a low-dimensional latent vector into a pathological speech spectrogram. A latent vector shown in Equation 9


$$\begin{array}{c} z\in {\mathbb{R}}^{100} \end{array}$$ (9)

was sampled from a uniform distribution over the range [–1,1]. The latent vector was projected through a fully connected layer, reshaping it to an initial feature tensor 8 × 40 × 512 for spectrogram synthesis that provides a small latent representation. The feature tensor was then progressively upsampled through four generator blocks to reach the desired spectrogram shape 128 × 640 × 1. Every generator block included a nearest neighbor resize operation to double the spatial resolution, followed by a 3 × 3 convolution layer with same padding and a Rectified Linear Unit (ReLU) activation function. The number of convolutional filters were lowered from 256 to 128 and then to 64 and finally to 1, refining the feature maps step-by-step while increasing the spatial resolution. The proposed architecture uses nearest-neighbor upsampling followed by convolution instead of the conventional generators which use transposed convolutions. This design achieves stable reconstruction of high-resolution time-frequency representations by reducing checkerboard artifacts from transposed convolution. The complete architecture of the generator is shown in Figure 2a. The output of the last layer was passed through a hyperbolic tangent (Tanh) activation function, which normalized the generated spectrogram values to the normalized range, producing a single-channel log-magnitude spectrogram that could be used for adversarial training and subsequent speech reconstruction.

**Figure 2:**
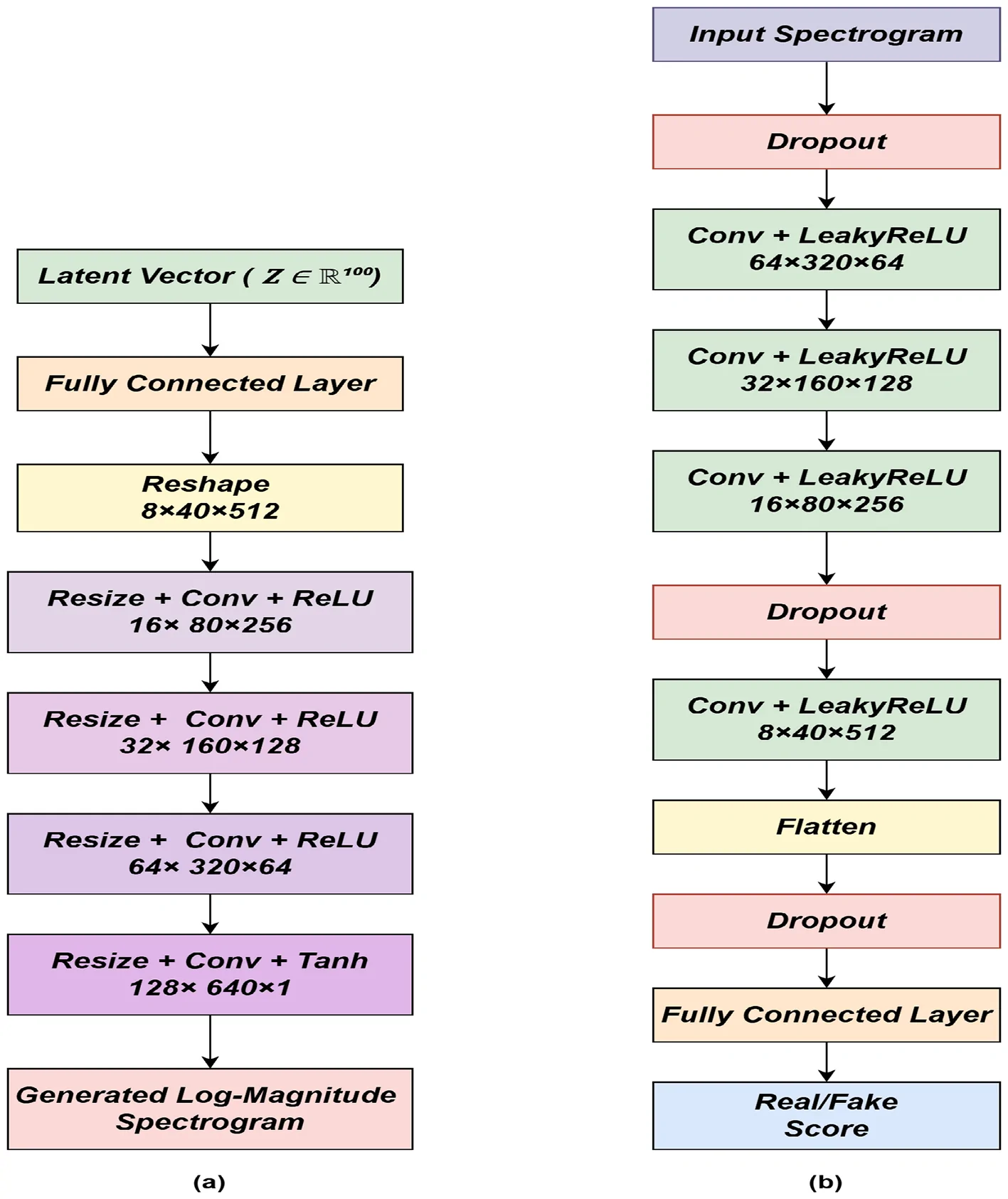
**(a)** Generator architecture used for pathological spectrogram synthesis. **(b)** Discriminator architecture for distinguishing real and generated pathological speech spectrograms.

#### 3.2.4 Discriminator architecture for speech

The discriminator was a deep convolutional classifier that was trained to distinguish between real and synthesized spectrograms. The network is composed of four convolutional layers of 64, 128, 256, and 512 filters respectively, with 4x4 kernels, a stride of two and the same padding. The discriminator model uses LeakyReLU activation functions with a negative slope coefficient of 0.2, which is typically used in GANs for discriminators in image and spectrogram tasks. Dropout regularization with a probability of 0.2 was implemented to increase generalization and prevent discriminator overfitting at the first, last convolution layers, and the output layer. The extracted feature maps were flattened and mapped to a single scalar output through a fully connected layer representing the probability that the input sample was real. The discriminator is a hierarchical convolutional feature extractor that progressively spatially down samples to distinguish real spectrograms from generated samples. The full architecture of the discriminator is shown in Figure 2b.

#### 3.2.5 Adversarial training strategy

The generator and discriminator were optimized simultaneously by Adam optimization. The generator learning rate was set to ηG = 10^−4^, and the discriminator learning rate was fixed to ηD = 10^−5^. Exponential decay parameters were set to β_1_ = 0.5 and β_2_ = 0.999, as the standard GAN training practice. The training was performed for 1,500 epochs with a mini-batch size of 16 samples. Binary cross-entropy loss was adopted for adversarial optimization. The discriminator loss is defined in Equation 10


$$\begin{array}{c} L_{D}=-\frac{1}{2}\left[E_{x\sim p_{\text{data}}}\log D\left(x\right)+E_{z\sim p_{z}}\log\left(1-D\left(G\left(z\right)\right)\right)\right] \end{array}$$ (10)

where *D*(·) and *G*(·) denote the discriminator and generator functions, respectively. Similarly, the generator objective is defined in Equation 11


$$\begin{array}{c} L_{G}=-E_{z\sim p_{z}}\log D\left(G\left(z\right)\right) \end{array}$$ (11)

Before the discriminator evaluation, both the real and synthesized spectrograms were normalized by their maximum absolute magnitude so that the discrimination is not sensitive to the scale. The full set of training and optimization parameters used in this study is listed in Table 3.

**Table 3:** GAN training hyperparameters.

Parameter	Value
Latent dimension	100
Batch size	16
Epochs	1500
Generator learning rate	10^−4^
Discriminator learning rate	10^−5^
Optimizer	Adam
**β_1_**	0.5
**β_2_**	0.999

#### 3.2.6 Spectrogram reconstruction and waveform synthesis

New latent vectors were sampled after training and fed into the generator to create synthetic spectrograms. Then, the inverse normalization procedure was applied using Equation 12


$$\begin{array}{c} S_{log}^{\prime }\left(k,m\right)=\left(3\cdot S_{G}\left(k,m\right)\right)\sigma_{k}+\mu_{k} \end{array}$$ (12)

where *S*_*G*_(*k, m*) is the output of the generator. The linear magnitude spectrum was obtained using Equation 13


$$\begin{array}{c} \exp\left(S_{\log}^{\prime }\left(k,m\right)\right)-10^{-6} \end{array}$$ (13)

To suppress frame-wise spectral fluctuations; a temporal smoothing was performed using a median filter of size 3 × 3. Since the GAN only generated magnitude information, the phase was estimated using iterative STFT magnitude inversion with gradient descent optimization using the L-BFGS optimizer. The reconstruction was performed with 200 iterations, with the same STFT parameters used in the analysis and synthesis. The whole reconstruction pipeline is shown in Figure 3. The reconstructed signal was fixed to 640 frames, and thus the total length of the reconstructed signal was 10.256 s. The reconstructed audio was also subjected to a short informal perceptual listening check, which indicated decent intelligibility of the sustained vowel and confirmed that the reconstructed signals were perceptually interpretable. This listening check was used only for qualitative inspection of the reconstructed audio and was not treated as an objective acoustic or perceptual validation measure. After reconstruction, the generated audio was separately post-processed using the MATLAB (MathWorks, Inc., Natick, Massachusetts, USA) detectSpeech function for voice activity detection. This post-processing was applied only to the reconstructed synthetic audio and was used to isolate the sustained phonation and trim the unvoiced and zero-padded regions introduced during preprocessing. Then, the post-processed waveform was used in cepstrogram computation for balancing the dataset for severity detection. Figure 4 shows a comparison between the original and post-processed synthesized pathological vowel waveforms and duration comparison of the original high severity and GGS audio is listed in Table 4. To check whether utterance duration could affect the classification results independently, the duration distributions of the real low-severity, real high-severity and GGS samples were analyzed. The duration-only analysis did not demonstrate reliable severity discrimination in the training data. The coefficient for duration in the logistic-regression is not statistically significant (*p* = 0.337). The threshold-based classifier is only at the level of the majority class. On average, the post-processed GGS samples were also shorter than the real high-severity training samples, indicating that they did not simply learn the duration distribution of the real high-severity class. Therefore, there was no evidence that the GGS classification performance was explained by duration as an explicit feature or by directly reproducing the duration characteristics of the real high-severity class. However, duration-related characteristics may still be indirectly reflected in the cepstrogram representation and cannot be completely excluded as a potential confounding factor. Thus, the present analysis does not establish that duration has no influence on the CNN predictions. The proposed framework was implemented in MATLAB within the Deep Learning Toolbox. Training was performed with mini-batch stochastic optimization, with GPU acceleration when available. Moreover, in the preprocessing stage, the parallel processing was also used to reduce the spectrogram extraction time for large datasets.

**Figure 3:**
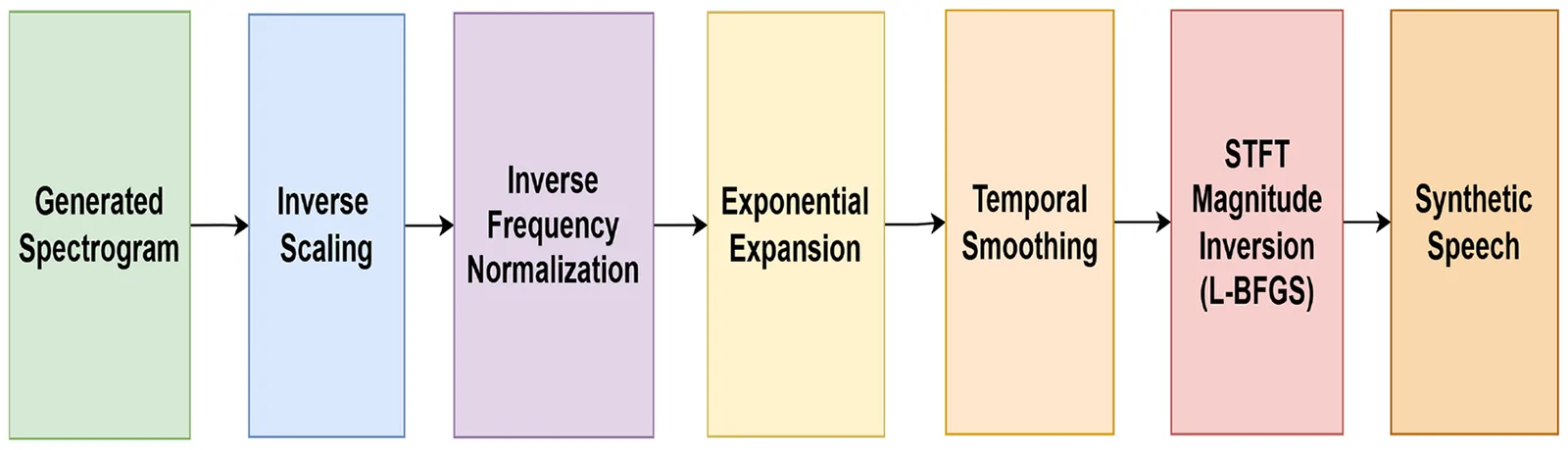
Reconstruction pipeline used for converting generated spectrogram magnitudes into time-domain pathological speech signals.

**Figure 4:**
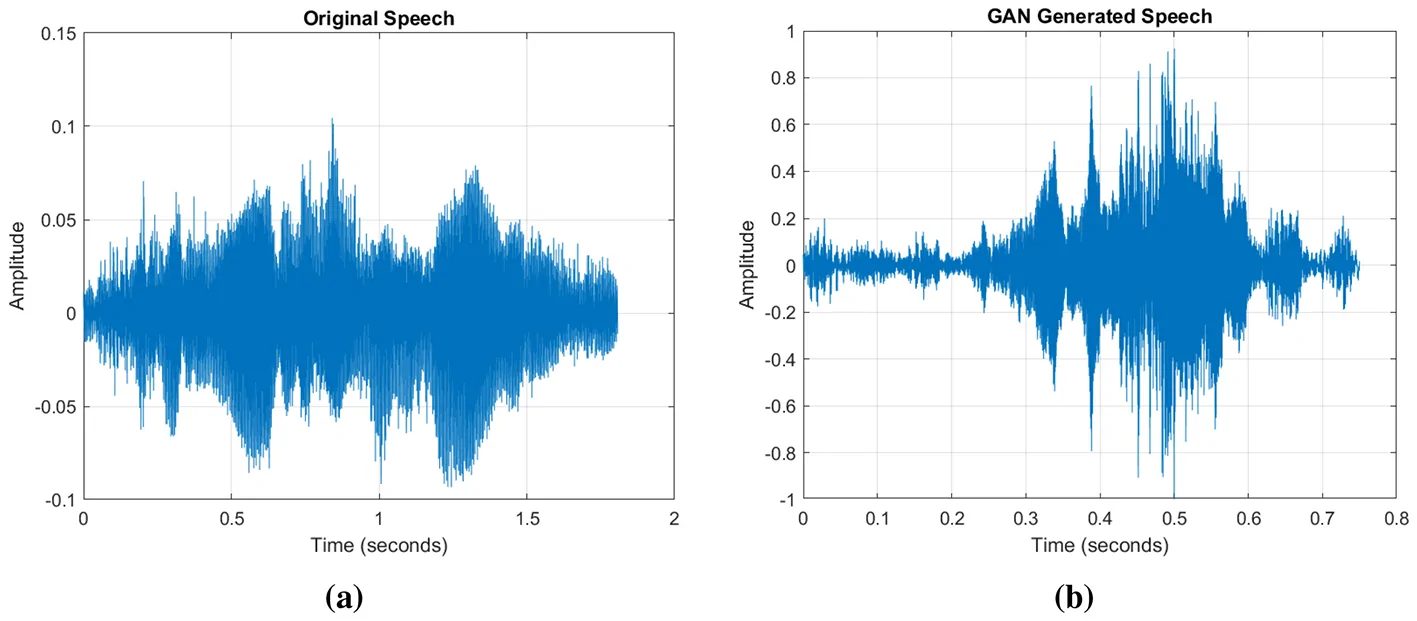
**(a)** Original speech. **(b)** Post-processed synthesized pathological vowel speech.

**Table 4:** Duration statistics of original high-severity and post-processed GGS audio.

Type	Mean (s)	SD (s)	Median (s)	Min (s)	Max (s)
Original high severity	2.7479	2.3180	1.8591	0.6211	17.9095
Post-processed GGS	1.3899	0.5267	1.2451	0.5252	3.0751

### 3.3 Cepstrogram computation

Speech signals were first converted to a single channel when stereo recordings were encountered, and then all signals were down-sampled to 8 kHz to ensure a common sampling rate before feature extraction. Short-time cepstral analysis was then performed using a 512-point Fourier transform, a 16 ms Hamming window and an 8 ms frame shift resulting from 50% overlap, consistent with the implementation used in the present work. The 16 ms window was selected to provide a short-time representation of the speech signal while retaining adequate temporal resolution for cepstral analysis, and the same window duration was consistently applied to all recordings to ensure uniform feature extraction across speakers. Let the speech signal in the frame be represented by *x*_*m*_[*n*], where *m* is the index of the frame. The real cepstrum of the *m*-th frame was calculated from the log-magnitude spectrum as shown in Equation 14


$$\begin{array}{c} c_{m}\left[q\right]=F^{-1}\left\{\log|F\left\{x_{m}\left[n\right]\right\}|\right\} \end{array}$$ (14)

where F{·} and $F^{-1}\left\{\cdot \right\}$ are the Fourier and inverse Fourier transforms, respectively (Oppenheim, 1969). This formulation is based on the standard homomorphic interpretation of speech, where convolutional source-filter components become additive in the cepstral domain after logarithmic spectral transformation (Oppenheim and Schafer, 2004). The frame-wise cepstral vectors were stacked over time to obtain a two-dimensional cepstrogram representation. Only the low-frequency cepstral region, that is, the first 128 cepstral bins of each frame, was used to produce the low-frequency cepstrogram used in the classification pipeline. In this study, the representation is termed a low-frequency cepstrogram. Technically, the retained cepstrals correspond to the low-quefrency region of the cepstrum. The low-frequency cepstrogram was chosen based on previous experiments in the same research line where the low-frequency cepstrogram was superior to other time-frequency representations (Aurobindo et al., 2025), and also because the low-frequency cepstral coefficients mainly encode slow-varying vocal-tract and spectral-envelope information, which is relevant for pathological speech analysis. The resulting low-frequency cepstrogram matrix was finally stored as an image and used as the input representation for severity detection. Such image-based speech representations have been increasingly used in pathological voice and Parkinsonian speech analysis because convolutional models can learn patterns in time-frequency image representation (Rahmatallah et al., 2025; Aurobindo et al., 2025).

### 3.4 Proposed WGAN-GP-based low frequency cepstrogram synthesis framework

In the proposed framework, a Wasserstein Generative Adversarial Network with Gradient Penalty (WGAN-GP) is employed to synthesize the pathological low frequency cepstrogram representations of each vowel separately, and the complete workflow of WGAN-GP is shown in Figure 5. Unlike the conventional GANs, which optimize the Jensen-Shannon divergence, WGAN-GP minimizes the Wasserstein distance between real and generated distributions, leading to better convergence behavior and training stability for limited biomedical datasets.

**Figure 5:**
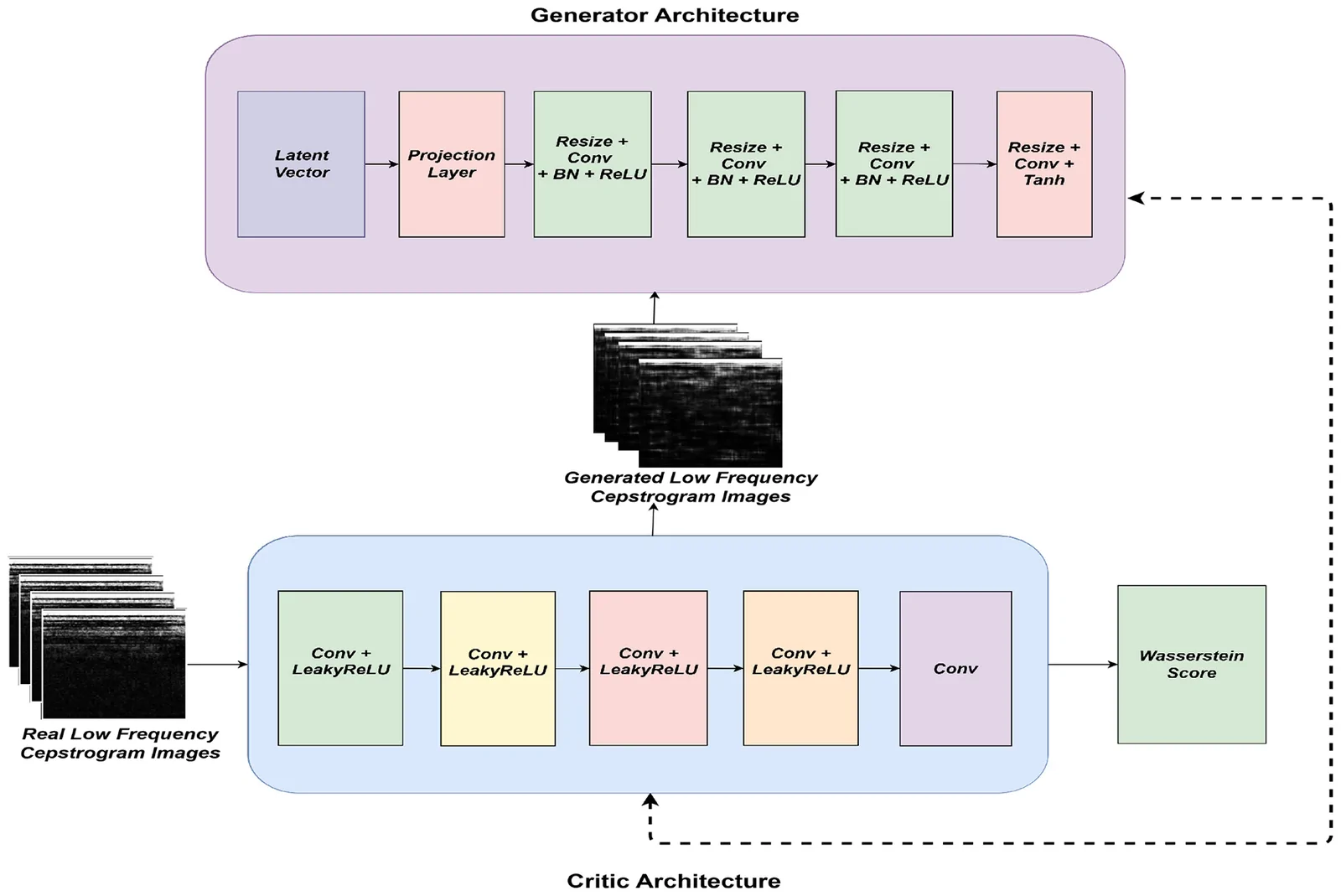
Overall architecture of the proposed WGAN-GP framework for pathological low-frequency cepstrogram synthesis.

#### 3.4.1 Low frequency cepstrogram preparation

The training samples were the low-frequency cepstrogram images extracted from sustained vowel recordings of Parkinsonian speakers. To ensure dimensional consistency for adversarial learning, all images were resized to a fixed spatial resolution of 128 × 128 pixels. Prior to the training, the pixel intensities were normalized to the interval [–1, 1] to be consistent with the output range of the generator activation function.

#### 3.4.2 Generator network

The generator learns to generate a synthetic low-frequency cepstrogram image from a low-dimensional latent representation via progressive feature expansion. A learnable projection layer projects a latent vector of dimension 100 sampled from a Gaussian distribution into a 8 × 8 × 512 feature tensor. Then, four resize-convolution blocks are used to learn hierarchical feature refinement and spatial upsampling. Each block is comprised of nearest-neighbor interpolation, convolution, batch normalization and ReLU activation and the parameters of the overall generator are listed in Table 5. Resize-convolutions were used instead of transposed convolutions to avoid checkerboard artifacts commonly found in image synthesis tasks. The last convolution layer is a hyperbolic tangent activation function to generate normalized gray-scale low-frequency cepstrogram images. The generator uses the nearest-neighbor interpolation + convolution operation to avoid the checkerboard artifacts of transposed convolution.

**Table 5:** Generator architecture.

Layer	Output size	Configuration
Input	100 × 1	Latent vector z~N(0,1)
Projection layer	8 × 8 × 512	Fully connected projection and reshape
Upsampling Block 1	16 × 16 × 256	Resize + Conv(5 × 5, 256) + BN + ReLU
Upsampling Block 2	32 × 32 × 128	Resize + Conv(5 × 5, 128) + BN + ReLU
Upsampling Block 3	64 × 64 × 64	Resize + Conv(5 × 5, 64) + BN + ReLU
Output layer	128 × 128 × 1	Resize + Conv(5 × 5, 1) + Tanh

#### 3.4.3 Critic network

The discriminator was replaced by a critic network that estimates the Wasserstein distance, rather than posterior probabilities. The critic takes as input either real or generated low frequency cepstrogram images and outputs an unconstrained scalar score that represents the realism of the sample. The architecture consists of four blocks of convolutional feature extractors with increasing spatial downsampling and LeakyReLU activations. Finally, a last convolution operation maps the learned feature representation to a single Wasserstein score. The overall critic parameters are summarized in Table 6. Batch normalization were intentionally not used in the critic unlike the standard GAN discriminator to preserve the Lipschitz continuity assumptions required for Wasserstein optimization with gradient penalty regularization. In the adversarial learning, the Wasserstein distance (Arjovsky et al., 2017) between the real and synthesized low-frequency cepstrogram distributions is minimized. The critic objective is defined in Equation 15


$$\begin{array}{c} L_{D}=E_{z\sim p_{z}}\left[D\left(G\left(z\right)\right)\right]-E_{x\sim p_{\text{data}}}\left[D\left(x\right)\right]+\lambda E_{\hat{x}\sim p_{\hat{x}}}\\ \;\left[{\left(||\nabla_{\hat{x}}D\left(\hat{x}\right){||}_{2}-1\right)}^{2}\right] \end{array}$$ (15)

where *D*(·) denotes the critic, *G*(·) represents the generator, and the final term corresponds to the gradient penalty that enforces the 1-Lipschitz constraint. The generator objective is formulated in Equation 16


$$\begin{array}{c} -\left[E\left[D\left(G\left(z\right)\right)\right]\right] \end{array}$$ (16)

that encourages the generator to maximize the critic score that is assigned to synthetic samples. Gradient penalty regularization stabilizes training and removes the need for weight clipping as used in early Wasserstein GANs.

**Table 6:** Critic architecture.

Layer	Output size	Configuration
Input	128 × 128 × 1	Low Frequency cepstrogram image
Conv Block 1	64 × 64 × 64	Conv(4 × 4, 64), stride 2, LeakyReLU
Conv Block 2	32 × 32 × 128	Conv(4 × 4, 128), stride 2, LeakyReLU
Conv Block 3	16 × 16 × 256	Conv(4 × 4, 256), stride 2, LeakyReLU
Conv Block 4	8 × 8 × 512	Conv(4 × 4, 512), stride 2, LeakyReLU
Output Layer	1 × 1 × 1	Conv(8 × 8, 1)

#### 3.4.4 Training strategy

The model was optimized with the Adam optimizer with a learning rate of 10^−5^, first-order momentum coefficient β_1_ = 0, and second-order momentum coefficient β_2_ = 0.9. The critic was trained five times for each generator update to properly estimate the Wasserstein distance during the optimization. The training was performed for 2,000 epochs with mini-batches of 32 low-frequency cepstrogram images. The coefficient of the gradient penalty was set to λ = 1 based on an ablation study comparing λ = 1, λ = 5, and λ = 10 which yielded final Wasserstein estimates of 0.8295, 1.0039, and 3.4578, respectively, indicating better Wasserstein-distance behavior for λ = 1. The Wasserstein distance of the ablation study is shown in Figure 6. This experimental result supports the selection of λ = 1, which is also consistent with previous work reporting that smaller gradient-penalty coefficients in the range of 1–5 can be effective for WGAN-GP image-generation networks, with λ = 1 providing better performance in the reported comparison (Ouyang et al., 2021). The training parameters are shown in Table 7. The Wasserstein-related training behavior was monitored throughout optimization to assess the stability and convergence of the adversarial training. After convergence, latent vectors were sampled from the prior distribution and fed to the trained generator to generate new low-frequency cepstrogram images. Figure 7 shows a comparison between the original low-frequency cepstrogram and a GAN-generated low-frequency cepstrogram image. The generated outputs were then rescaled to the intensity range of the original image and stored as grayscale images to be used in data augmentation and class balancing experiments with pathological speech classification models.

**Figure 6:**
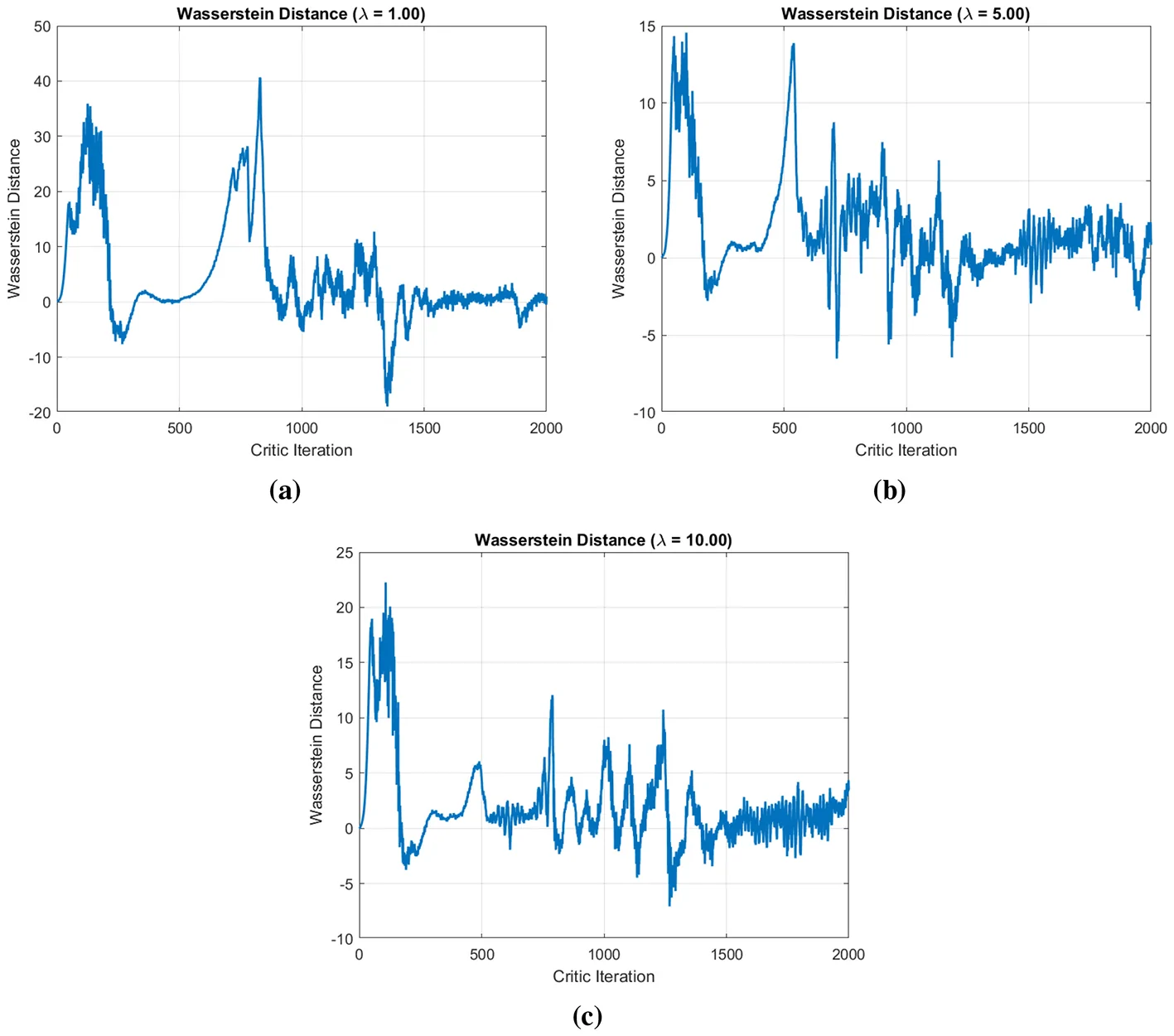
Wasserstein distance for different values of the gradient-penalty coefficient λ: **(a)** λ = 1, **(b)** λ = 5, and **(c)** λ = 10.

**Table 7:** Training hyperparameters.

Parameter	Value
Latent dimension	100
Input image size	128 × 128
Batch size	32
Epochs	2, 000
Learning rate	10^−5^
Optimizer	Adam

**Figure 7:**
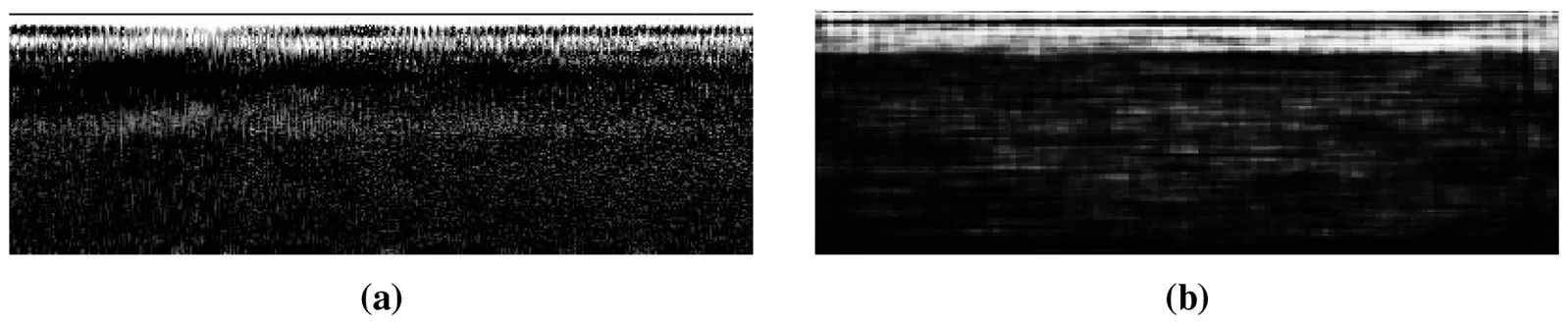
**(a)** Original speech low-frequency cepstrogram. **(b)** Synthesized pathological vowel low-frequency cepstrogram.

### 3.5 Proposed CNN model for severity detection

All the low-frequency cepstrogram images were resized to 128 × 128 pixels before being fed into the network to maintain a uniform input dimension. The proposed network contains four convolutional blocks. Each block consists of a 3 × 3 convolutional layer with 64, 128, 256, and 512 filters, batch normalization, the ReLU activation function and 2 × 2 max-pooling. These layers learned spectral patterns of increasing abstraction while decreasing the spatial resolution of the feature maps. Then the extracted features were flattened and fed into two fully connected layers with 128 and 64 neurons.Dropout layers were added with a rate of 0.5 after the fully connected layers to avoid overfitting. The last classification layer had the same number of neurons as the number of severity classes with a softmax layer to yield the class probabilities. Training was done with Adam optimizer, with initial learning rate 1 × 10^−4^, mini-batch size 32 and 35 epochs. The performance was monitored during training with the validation set. All experiments were conducted using MATLAB R2024b on an Acer Predator Helios system equipped with an Intel Core i7-14700HX processor and an NVIDIA (NVIDIA Corporation, Santa Clara, California, USA) GeForce RTX 4050 graphics processing unit (GPU) with 6 GB of VRAM. Computations were run on a GPU for speed. The model performance was evaluated on the test set using accuracy, precision, recall, specificity and F1-score from the confusion matrix. Furthermore, Grad-CAM was used to visualize the most influential cepstrogram regions for the model predictions, providing interpretability to the learned discriminative features.

## 4 Results and discussion

The main aim of this work was to compare two GAN-based augmentation methods, namely GAN-generated speech (GGS) and GAN-generated image (GGI), for reducing class imbalance in low-frequency cepstrogram-based severity classification. The original dataset was used as a baseline reference for the comparison of the models and had an imbalanced distribution of 300 high-severity samples and 450 low-severity samples with speakers in both classes producing three utterances of each vowel across 50 speakers. Such class imbalance is common in clinical speech and medical classification tasks, where minority or severe classes may be under-represented and may influence model training and the representation of minority classes (Rana et al., 2022; Zhou et al., 2026; Huang and Jafari, 2023). For the SI evaluation, all 15 utterances belonging to a given speaker, corresponding to three repetitions of each of the five sustained vowels, were kept within the same data partition. Thus, no speaker contributed utterances to more than one of the training, validation, or testing sets. As shown in Table 8, the training set for the SI consisted of 11 high-severity and 21 low-severity speakers, the validation set consisted of five high-severity and five low-severity speakers, and the test set consisted of four high-severity and four low-severity speakers. Consequently, the SI test set contained 120 utterances contributed by eight independent speakers. In the SD evaluation, the utterances were split at the utterance level rather than at the speaker level. Thus, the same speaker may have utterances in the training, validation and testing sets. The speaker overlap is intentional and enables the SD protocol to evaluate the model performance under the speaker-shared condition, compared to the speaker-disjoint SI protocol. In both augmentation methods, 150 synthetic high-severity samples were generated using the GAN trained only on the training-partition samples of the speaker-independent task to increase the minority class from 300 to 450 samples overall. The synthetic samples were not included in the validation or testing sets of either task. The original dataset partitions were reassigned before the final baseline and augmentation experiments to maintain consistent evaluation conditions across the experimental settings. For the speaker-independent task, 30 real high-severity samples were reassigned from the original training set to validation and 15 real high-severity samples were reassigned to testing, resulting in 165 real high-severity samples in training, 75 in validation, and 60 in testing. The corresponding low-severity samples were retained, resulting in 315, 75, and 60 low-severity samples in the training, validation, and testing partitions, respectively. Thus, the speaker-independent task comprised 480 training samples, 150 validation samples and 120 testing samples, with disjoint speaker IDs in the three partitions. Therefore, the GAN generators did not have access to any speaker from the validation or testing partitions. The resulting validation and testing partitions were kept fixed across all experimental conditions, and the original dataset baseline, GGS-augmented and GGI-augmented models were evaluated using the same real validation and testing samples. Thus, the synthetic samples were used exclusively for training, while the validation and testing sets remained entirely real and identical across the three speaker-independent conditions. The same 150 synthetic samples were also used to augment the training set of the speaker-dependent task. All speakers whose real utterances were used to train the GAN were also represented by real utterances in the speaker-dependent training partition. Since the speaker-dependent protocol was constructed using an utterance-level split rather than a speaker-level split, the same speakers could contribute utterances to the training, validation, and testing partitions by design. In this setting, 23 real high-severity utterances were reassigned to the validation set and 22 real high-severity utterances were reassigned to the testing set. Consequently, the final augmented training set contained 315 high-severity and 315 low-severity samples, while the validation and testing sets contained 75 and 60 samples per class for the speaker-independent task and 68 and 67 samples per class for the speaker-dependent task, respectively, and consisted entirely of real samples. The details of the resulting dataset composition are listed in Table 9. Accordingly, GGS and GGI were evaluated as two complete augmentation pipelines rather than as a controlled experiment isolating the effect of augmentation domain. Differences in performance may therefore reflect both the stage at which augmentation is applied and the corresponding GAN architecture, objective function, resolution, and training configuration. The GGS and GGI conditions used class-balanced datasets, with synthetic samples included only in the training sets, allowing the two augmentation approaches to be compared under the same class-balanced evaluation framework. Prior work has demonstrated the utility of GAN-based augmentation in the setting of minority class balancing, as it can generate realistic samples while maintaining the data structure pertinent to the task (Rana et al., 2022; Huang and Jafari, 2023; Chatziagapi et al., 2019). The comparison was carried out under both speaker-independent and speaker-dependent evaluation methods, to evaluate if the effectiveness of augmentation is affected by speaker variability. This distinction is relevant as speaker-independent settings are generally more difficult due to the need of the model to generalize to speaker-dependent acoustic characteristics, whereas speaker-dependent settings can rely on shared speaker information between training and testing (Chatziagapi et al., 2019; Javanmardi et al., 2024a).

**Table 8:** Speaker distribution across the training, validation, and testing split for the speaker-independent evaluation.

Data split	High severity	Low severity
Training set	11 speakers AVPEPUDEA00: 03, 10, 11, 14, 46 48, 51, 53, 55, 57, 59	21 speakers AVPEPUDEA00: 01, 02, 05, 06, 07, 08, 09, 13, 15, 16, 17, 38, 42, 45, 47, 49, 50, 52, 54, 56, 58
Validation set	5 speakers AVPEPUDEA00: 30, 31, 35, 37, 39	5 speakers AVPEPUDEA00: 23, 26, 27, 29, 32
Test set	4 speakers AVPEPUDEA00: 24, 25, 41, 43	4 speakers AVPEPUDEA00: 20, 21, 22, 34
Total	20 speakers	30 speakers

**Table 9:** Composition of real and synthetic samples for speaker-independent and speaker-dependent PD hypokinetic dysarthria severity detection.

Dataset split	Speaker-independent			Speaker-dependent		
	High severity		Low severity	High severity		Low severity
	Real	Synthetic	Real	Real	Synthetic	Real
Original dataset (without GAN)						
Training	165 (210–30–15)	0	315	165 (210–23–22)	0	315
Validation	75 (45+30)	0	75	68 (45+23)	0	68
Testing	60 (45+15)	0	60	67 (45+22)	0	67
Total	300	0	450	300	0	450
GAN-augmented dataset						
Training	165	150	315	165	150	315
Validation	75	0	75	68	0	68
Testing	60	0	60	67	0	67
Total	300	150	450	300	150	450

### 4.1 Speaker independent evaluation

#### 4.1.1 Original dataset baseline

The speaker-independent baseline experiment on the original low-frequency cepstrogram dataset achieved a total test accuracy of 48.33%, from the Figure 8 confusion matrix showing 31 high-severity samples and 27 low-severity samples correctly classified. However, the model had a lot of confusion between classes, misclassifying 29 high-severity samples as low-severity and 33 low-severity samples as high-severity. The single-split speaker-independent baseline accuracy of 48.33% was slightly below the chance-level reference accuracy of 50.00%, obtained by always predicting either class in the balanced test set. This indicates that the single-split baseline performance was sensitive to the particular data partition. Therefore, the single-split result was not considered sufficient to determine the effectiveness of augmentation, and the baseline and augmented conditions were further evaluated using repeated speaker-stratified five-fold cross-validation with five independent random seeds. The findings demonstrate the difficulties of learning speaker-invariant pathological attributes from an imbalanced dataset. In pathological voice and speech classification studies, similar limitations have been reported where inadequate representation of the minority class leads to poor generalization and increased misclassification of clinically important categories (Zhou et al., 2026; Javanmardi et al., 2024a). The model achieved a training accuracy of 81.25% but a validation accuracy of only 58.67% as shown in Figure 9, indicating a considerable gap between training and validation performance and limited generalization ability to unseen speakers, and overall test metrics are listed in Table 10. This kind of performance limitation is often seen in deep CNN models trained on small or imbalanced datasets, as the network tends to memorize dominant patterns instead of learning class-invariant discriminative features (Rana et al., 2022; Zhou et al., 2026). The corresponding precision, recall, specificity, and F1-score were 48.44%, 51.67%, 45.00%, and 50.00%, respectively. This baseline thus serves as a reference to evaluate the effectiveness of GGS and GGI in handling data imbalance. The original speaker-independent baseline with speaker-level analysis as shown in Table 11 achieved an accuracy of 62.50%, precision of 60.00%, recall of 75.00%, specificity of 50.00% and F1-score of 66.67%. Speaker-level predictions were obtained using majority voting across the utterance-level predictions for each speaker. The results indicate moderate performance for speaker-level classification, with higher recall than specificity, indicating that the model was better at correctly identifying high-severity speakers than low-severity speakers. The relatively lower specificity suggests that some low-severity speakers were incorrectly classified as high severity. These results further underline the variation of model performance across speakers and support the need to assess augmentation methods in a speaker-independent setting.

**Figure 8:**
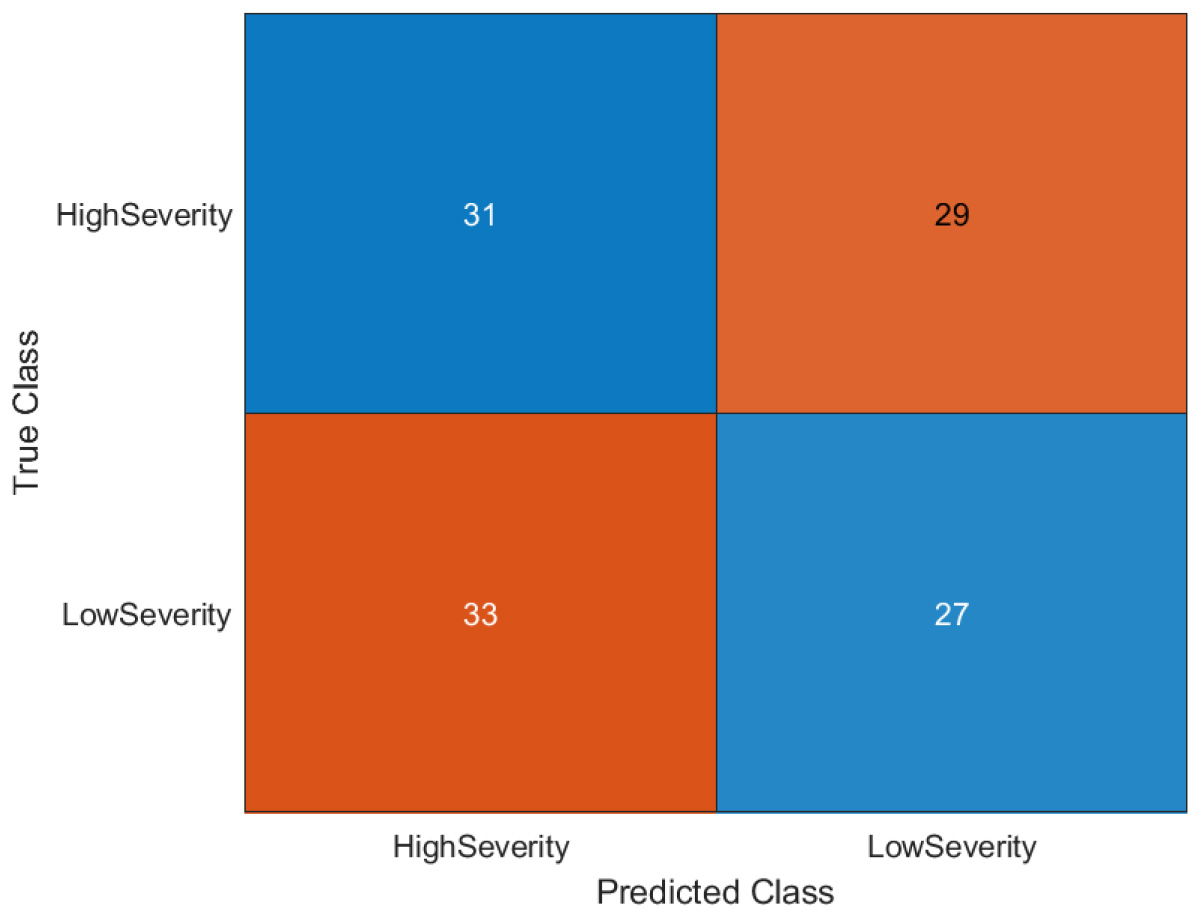
Confusion matrix for speaker-independent severity detection in original dataset.

**Figure 9:**
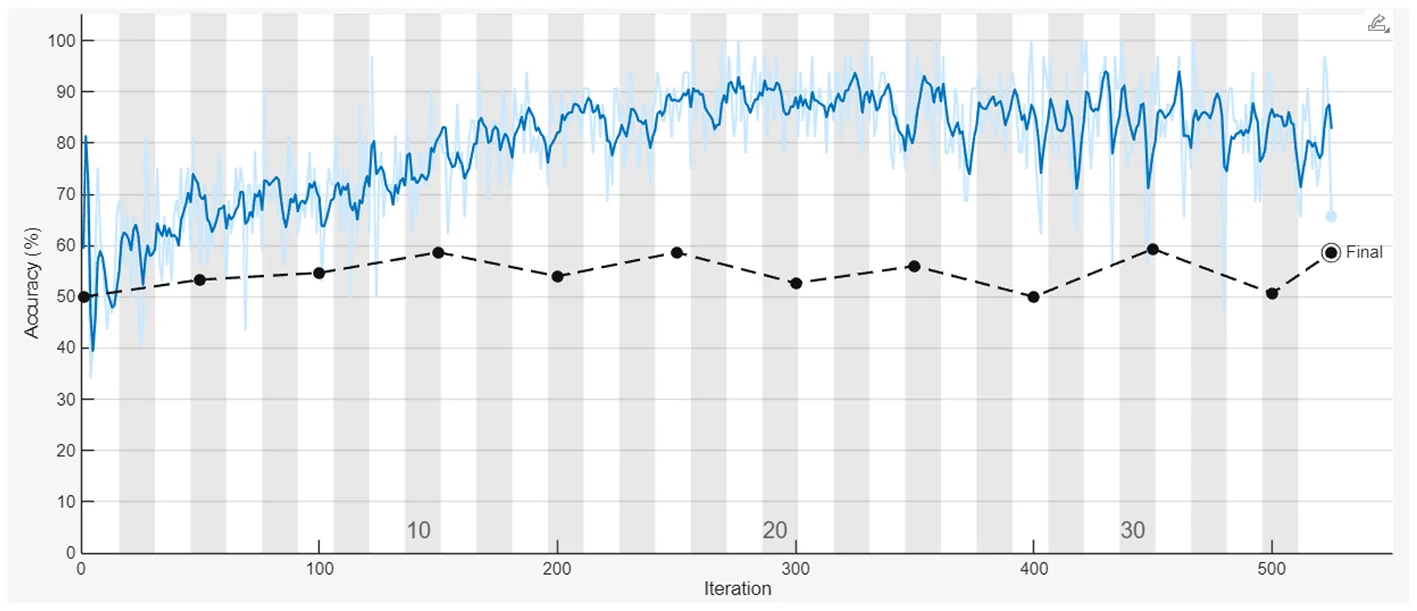
Training accuracy (Blue) and Validation accuracy (Dotted) for speaker-independent severity detection in original dataset.

**Table 10:** Baseline performance using the original speaker-independent low-frequency cepstrogram dataset.

Metrics	Original dataset
Training accuracy	81.25%
Validation accuracy	58.67%
Test accuracy	48.33%
Precision	48.44%
Recall	51.67%
Specificity	45.00%
F1-score	50.00%

**Table 11:** Speaker-level performance of the original speaker-independent baseline.

Metric	Speaker-level performance
Accuracy	62.50%
Precision	60.00%
Recall	75.00%
Specificity	50.00%
F1-Score	66.67%

#### 4.1.2 GAN-generated speech vs. GAN-generated image for data imbalance mitigation

The GAN-generated speech augmentation has increased the class distribution by generating an equal number of samples for the minority class and the achieved training accuracy is 93.75% and validation accuracy is 65.33% as shown in Figure 10 and the test accuracy achieved is 59.17%, which is an improvement over the original dataset. The model achieved 60.78% precision, 51.67% recall, 66.67% specificity and F1-score of 55.86%, and the confusion matrix for the GAN-generated speech augmentation is shown in Figure 11. This behavior is consistent with prior GAN-based augmentation studies in speech classification, where the generation of synthetic minority classes can improve the representation of the minority class and influence the balance between sensitivity and specificity (Chatziagapi et al., 2019). GGS correctly identified 31 high-severity samples and 40 low-severity samples, as shown in the confusion matrix. However, the model misclassified 29 samples of high severity as low severity, and 20 samples of low severity as high severity. This shows that the model still has difficulties in distinguishing between the two severity classes. The specificity of 66.67% from this model implies that it was relatively good at identifying the low severity samples. The recall of 51.67% means that about half of the high severity samples were correctly identified. The GGS improved test accuracy from 48.33% to 59.17%, precision from 48.44% to 60.78% and specificity from 45.00% to 66.67%, F1 score from 50.00% to 55.86% compared to the original dataset, while the recall remained unchanged at 51.67%. Such changes in sensitivity and specificity are commonly observed in studies focused on class-imbalance mitigation, especially when augmentation modifies the decision boundary between minority and majority classes (Zhou et al., 2026; Huang and Jafari, 2023). As shown in Table 12, the speaker-level analysis of the GAN-generated speech augmentation yielded an accuracy of 62.50%, precision of 66.67%, recall of 50.00%, specificity of 75.00%, and F1-score of 57.14%. The higher specificity indicates that GGS was better at correctly identifying the low-severity speakers, while the lower recall indicates that it was more difficult to correctly identify the high-severity speakers at the speaker level. These results suggest that GGS resulted in a more conservative speaker-level classification with fewer low severity speakers being classified as high severity.

**Figure 10:**
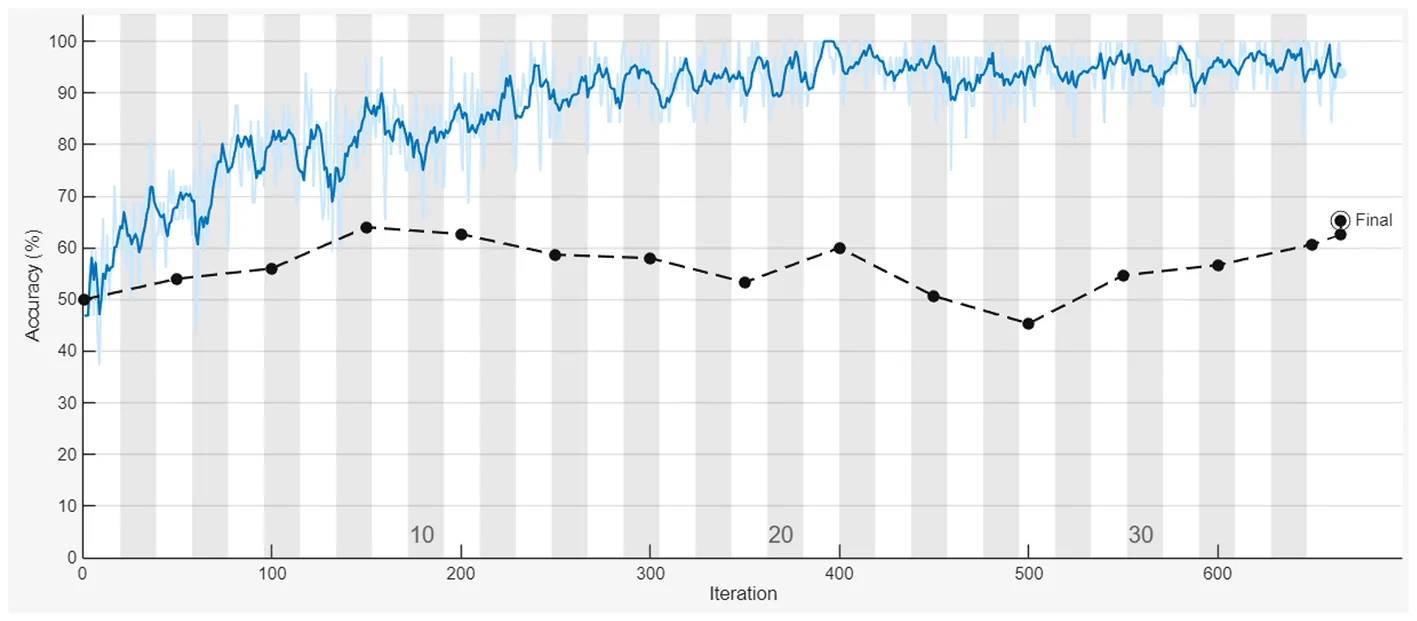
Training accuracy (Blue) and Validation accuracy (Dotted) for speaker-independent severity detection in GAN-generated speech augmentation.

**Figure 11:**
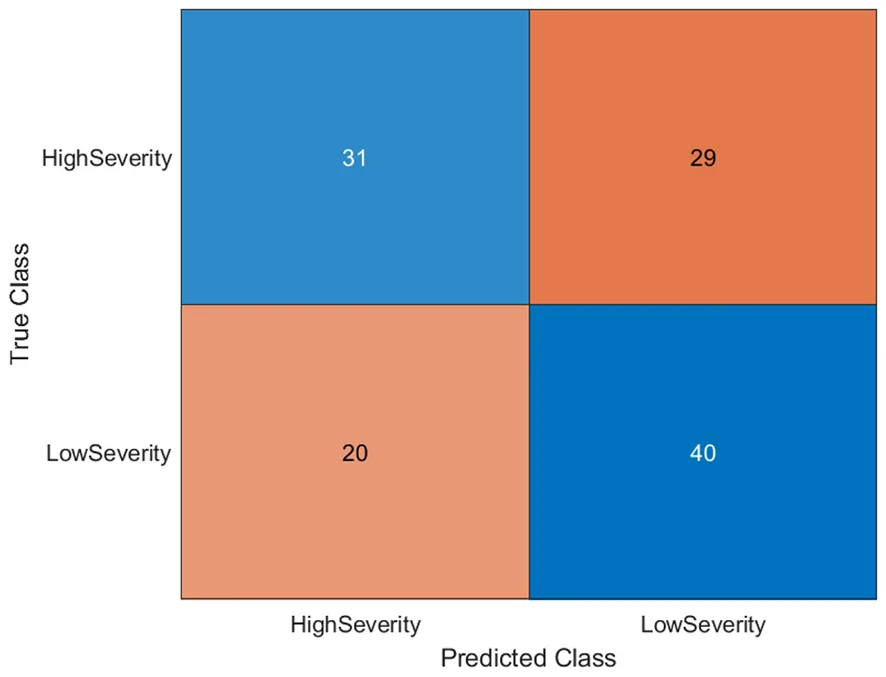
Confusion matrix for speaker-independent severity detection in GAN-generated speech augmentation.

**Table 12:** Speaker-level performance comparison of GGS and GGI under speaker-independent evaluation.

Metric	GAN-generated speech (GGS)	GAN-generated image (GGI)
Accuracy	62.50%	62.50%
Precision	66.67%	60.00%
Recall	50.00%	75.00%
Specificity	75.00%	50.00%
F1-score	57.14%	66.67%

In contrast, the best speaker-independent performance was achieved using the GAN-generated image augmentation, with a test accuracy of 61.67%, a validation accuracy of 66.67%, training accuracy of 96.87% as depicted in Figure 12, and a precision of 63.46%. Recall increased to 55.00%, while GGI improved specificity to 68.33% and achieved an F1-score of 58.93%. These differences can also be seen in the confusion matrix as shown in Figure 13. GGI correctly identified 33 high-severity samples and 41 low-severity samples, while misclassifying 27 high-severity samples as low severity and 19 low-severity samples as high severity. This indicates that the GGI configuration produced a slightly more balanced classification outcome, with improvements in test accuracy, precision, recall, specificity, and F1-score compared with the original dataset. Compared with GGS, GGI also achieved higher test accuracy, precision, recall, specificity, and F1-score. One possible explanation is that augmentation on cepstrogram images directly preserves more local time-frequency texture patterns that CNNs use for classification than generating waveforms and then converting them to cepstrograms (Chatziagapi et al., 2019; Javanmardi et al., 2024a). As shown in Table 13, GGI achieved higher observed sample-level values than GGS across all reported evaluation metrics, although the differences were relatively modest for recall and specificity. The higher accuracy and specificity observed with GGI in the single-split speaker-independent evaluation suggest more favorable performance on unseen speakers, as speaker-independent evaluation provides a more stringent assessment of generalization under conditions that more closely reflect practical deployment. In real-world clinical decision support systems, higher specificity is desirable because it lowers false alarm rates and improves confidence in the outputs of automated screening (Zhou et al., 2026). As shown in Table 12, the speaker-level analysis of the GAN-generated image augmentation also achieved an accuracy of 62.50%, with a precision of 60.00%, recall of 75.00%, specificity of 50.00%, and F1-score of 66.67%. The higher recall indicates that GGI was more effective in correctly identifying high-severity speakers, although this was accompanied by lower specificity and increased misclassification of low-severity speakers as high severity. The higher F1-score of GGI also indicates a better balance between precision and recall at the speaker level. Although both augmentation methods achieved the same speaker-level accuracy, their performance differed in terms of the balance between high-severity and low-severity speaker identification.

**Figure 12:**
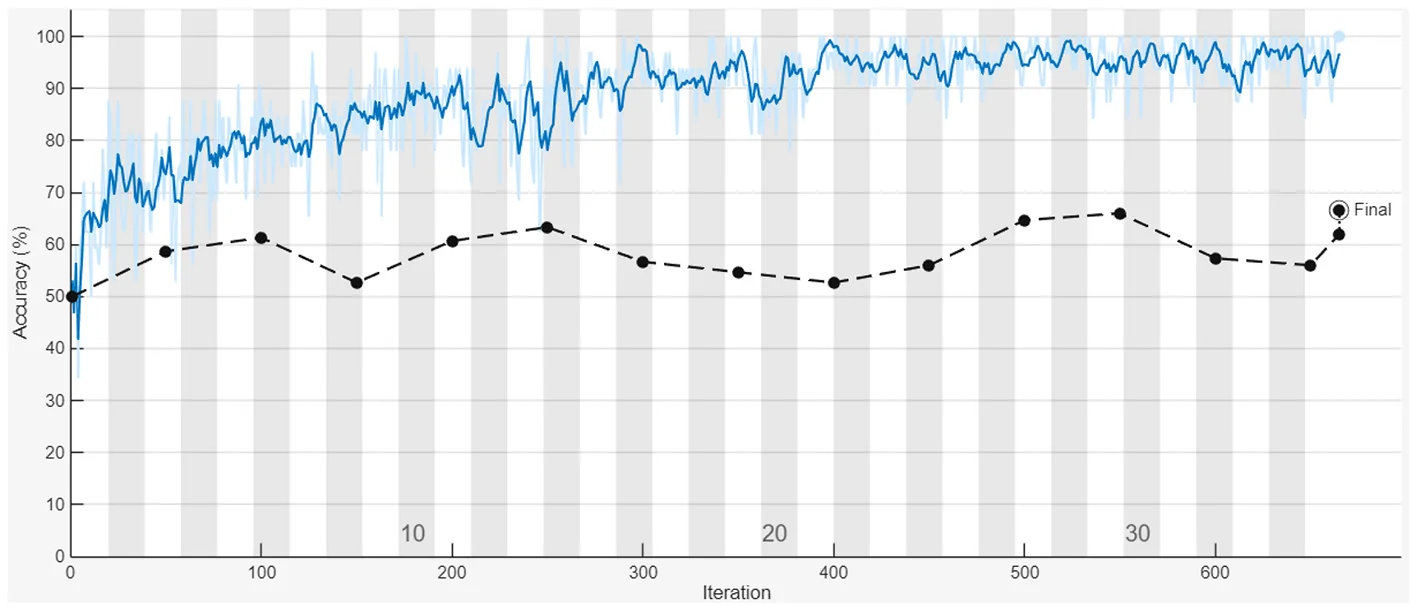
Training accuracy (Blue) and Validation accuracy (Dotted) for speaker-independent severity detection in GAN-generated image augmentation.

**Figure 13:**
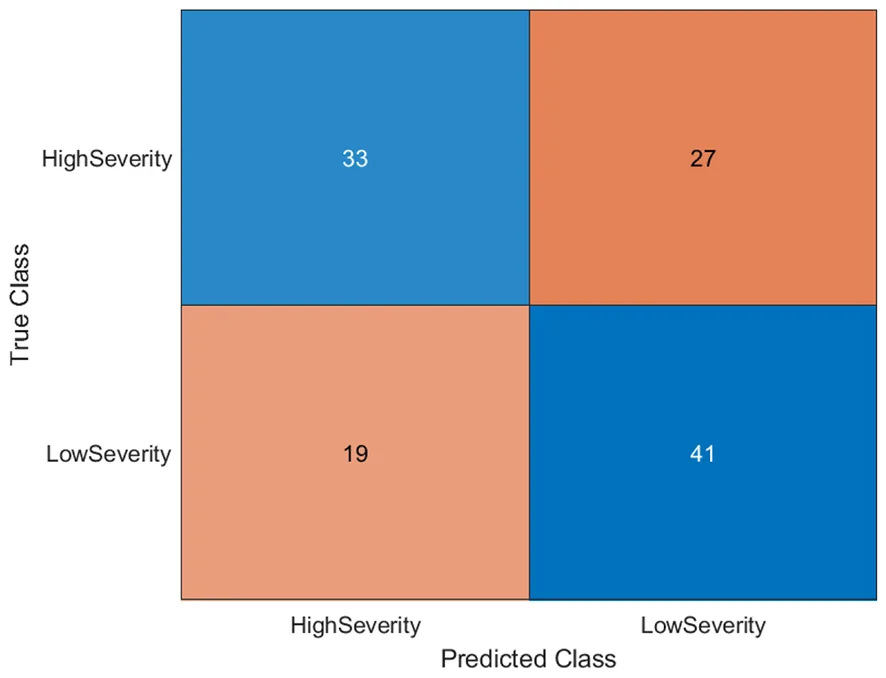
Confusion matrix for speaker-independent severity detection in GAN-generated image augmentation.

**Table 13:** Performance comparison of augmentation methods under speaker-independent evaluation.

Metric	GAN-generated speech	GAN-generated image
Training accuracy	93.75%	96.87%
Validation accuracy	65.33%	66.67%
Test accuracy	59.17%	61.67%
Precision	60.78%	63.46%
Recall	51.67%	55.00%
Specificity	66.67%	68.33%
F1-score	55.86%	58.93%

To assess the robustness of the speaker-independent results, five-fold speaker-stratified cross-validation was repeated using five independent random seeds, resulting in 25 fold-seed evaluations for each experimental condition, as shown in Table 14. For each fold-seed evaluation, the trained model for each experimental condition in Baseline, GGS, and GGI was evaluated on the same fixed speaker-independent test set. Thus, the composition of the test set remained unchanged across all 25 fold-seed evaluations, while the training/validation partition varied according to the speaker-stratified folds and random seed. The in-fold validation split was retained as a training and monitoring signal, but was not used to calculate the reported test accuracy. The synthetic samples used in these repeated evaluations were generated once from the designated speaker-independent training partition and were subsequently reused across the fold-seed evaluations. This analysis should be seen as a repeated robustness analysis of the classifier considering different speaker-stratified partitions and random initializations, rather than a fully nested evaluation in which GAN training and synthetic-sample generation are repeated independently in each cross-validation fold. The repeated evaluation was therefore not intended to provide an independent estimate of the variability of the complete GAN-generation pipeline. The main objective of the statistical analysis was to determine whether classification performance differed between GAN-augmented and original-data conditions. The baseline obtained an average test accuracy of 53.13% ± 3.26% over the 25 fold-seed evaluations on the original dataset. The corresponding augmented models obtained mean test accuracies of 56.47% ± 3.40% for GGS and 55.47% ± 3.35% for GGI. Both augmentation methods achieved a higher mean accuracy than the original baseline, with the highest mean accuracy for GGS. This repeated evaluation also confirms that the mean accuracy obtained exceeds the majority-class rate (i.e., 50%) for all three conditions. The improvement was also visible in the other classification measures. GGS had higher precision, recall, specificity and F1-score compared to the baseline. GGI had higher precision, recall and F1-score compared to the baseline, while its specificity was slightly lower than the baseline. The standard deviations across the 25 fold-seed evaluations show variation across cross-validation partitions and random initializations. The mean accuracies for both augmentation strategies were higher than the original baseline, but the improvements were relatively small compared with the variability across the repeated evaluations. Hence, the performance of the augmentation methods was evaluated based on the mean performance and the variance in performance across different data splits and random initializations. Thus, McNemar's exact test was performed on the paired predictions of the original-dataset baseline and GGS separately from the paired predictions of the original-dataset baseline and GGI. The comparison of GGS and GGI was not treated as a statistical hypothesis test but as a comparative performance analysis based on the reported classification metrics and repeated fold-seed evaluations, as shown in Table 15. For GGS, 204 samples were correctly classified by the baseline but incorrectly classified by GGS, whereas 304 samples were incorrectly classified by the baseline but correctly classified by GGS (*b* = 204, *c* = 304, and *p* = 1.054 × 10^−5^). For GGI, 239 samples were correctly classified by the baseline but incorrectly classified by GGI, whereas 309 samples were incorrectly classified by the baseline but correctly classified by GGI (*b* = 239, *c* = 309, and *p* = 0.0031675). These pooled McNemar analyses indicate systematic changes in classification decisions between each augmented condition and its corresponding original-dataset baseline. However, because the same fixed test samples were evaluated repeatedly across the 25 fold-seed evaluations, pooling all predictions does not provide independent observations and may yield anticonservative significance estimates. Therefore, the pooled McNemar results are treated as supplementary evidence of changes in classification decisions rather than as the primary significance analysis. To address this dependence, paired *t*-tests were additionally performed within each speaker-stratified fold across the five independent seeds using the same fixed real test set as shown in Table 16. For GGS, significantly higher accuracy than the baseline was observed in folds 1 (*p* = 0.042), 2 (*p* = 0.036), and 3 (*p* = 0.033), whereas the differences were not statistically significant in folds 4 and 5. For GGI, significantly higher accuracy than the baseline was observed only in fold 1 (*p* = 0.022), while the remaining folds showed no statistically significant differences. These fold-level analyses provide a more appropriate assessment of the consistency of augmentation effects across the speaker-stratified training partitions without treating the repeated predictions of the same test samples as independent observations. This repeated evaluation also confirms the mean accuracy obtained exceeds the majority-class rate(i.e., 50%).

**Table 14:** Speaker-independent performance across repeated fold-seed evaluations.

Performance measure	Original dataset	GGS	GGI
Accuracy (%)	53.13 ± 3.26	56.47 ± 3.40	55.47 ± 3.35
Precision	55.90 ± 0.98	65.30 ± 1.11	60.00 ± 0.94
Recall	25.50 ± 1.30	31.30 ± 0.89	33.10 ± 1.16
Specificity	80.70 ± 1.04	81.70 ± 0.95	77.90 ± 0.92
F1-score	33.30 ± 1.39	41.11 ± 0.76	41.15 ± 1.09

**Table 15:** McNemar's exact test for paired speaker-independent predictions.

Comparison	Baseline correct/ augmented model Incorrect (*b*)	Baseline incorrect / augmented model Correct (*c*)	Exact *p*-value	Significance
Original dataset vs. GGS	204	304	1.054 × 10^−5^	Significant
Original dataset vs. GGI	239	309	0.0031675	Significant

**Table 16:** Per-fold paired *t*-test results for the speaker-independent evaluation.

Fold	Original dataset vs. GGS	original dataset vs. GGI
1	**Significant**, *p* = 0.0416	**Significant**, *p* = 0.0222
2	**Significant**, *p* = 0.0360	Not significant, *p* = 0.5203
3	**Significant**, *p* = 0.0329	Not significant, *p* = 0.8907
4	Not significant, *p* = 0.5291	Not significant, *p* = 0.0801
5	Not significant, *p* = 0.4007	Not significant, *p* = 0.3950

Bold values indicate statistically significant differences between the compared methods (*p* < 0.05), based on paired *t*-tests.

### 4.2 Speaker-dependent evaluation

#### 4.2.1 Original dataset baseline

It is observed that the performance of the speaker-dependent classification in the baseline experiment is better than the SI experiment. The test accuracy of 66.42% is achieved with the precision, recall, specificity, and F1-score values of 92.31%, 35.82%, 97.01%, and 51.61%, respectively, and overall test metrics are listed in Table 17 and the training vs. validation accuracy is shown in Figure 14. The confusion matrix shown in Figure 15 revealed that 24 out of 67 high-severity samples were correctly identified. This reflects a high specificity toward low-severity cases, while the relatively lower recall indicates difficulty in correctly identifying high-severity samples. Since the speaker-dependent setting allows speaker-related acoustic characteristics to be shared across the training and testing datasets, the model can utilize speaker-specific information during classification. This difference in performance between SI and SD evaluation is in line with previous studies that have reported better performance of models when speaker-related acoustic characteristics are shared between training and testing samples (Chatziagapi et al., 2019; Javanmardi et al., 2024a). The improved performance in specificity shows the usefulness of speaker-specific acoustic information for low-frequency cepstrogram-based severity classification. This might overestimate performance compared to a real-world deployment but is still useful to understand the upper-bound behavior of the classifier in controlled speaker conditions (Javanmardi et al., 2024a). As shown in Table 18, the speaker-level analysis of the original speaker-dependent baseline achieved an accuracy of 86.96%, with precision, recall, specificity, and F1-score values of 100.00%, 66.67%, 100.00%, and 80.00%, respectively. The results show that the model correctly identified all low-severity speakers, as indicated by the specificity of 100.00%, while the recall of 66.67% indicates that some high-severity speakers were not correctly identified. The precision of 100.00% indicates that all speakers predicted as high severity were actually high severity. The speaker-level F1-score of 80.00% reflects a balance between precision and recall. These results further demonstrate the effect of speaker-related acoustic characteristics in the speaker-dependent setting, where information from the same speakers can be shared across the training, validation, and testing partitions. However, the difference between the sample-level and speaker-level performance also indicates that aggregation of predictions at the speaker level can produce different classification outcomes from individual utterance-level predictions. This speaker-level analysis provides an additional perspective on the performance of the baseline model under controlled speaker-dependent conditions.

**Table 17:** Baseline performance using the original speaker-dependent low-frequency cepstrogram dataset.

Metric	Original dataset
Training accuracy	90.25%
Validation accuracy	64.71%
Test accuracy	66.42%
Precision	92.31%
Recall	35.82%
Specificity	97.01%
F1-score	51.61%

**Figure 14:**
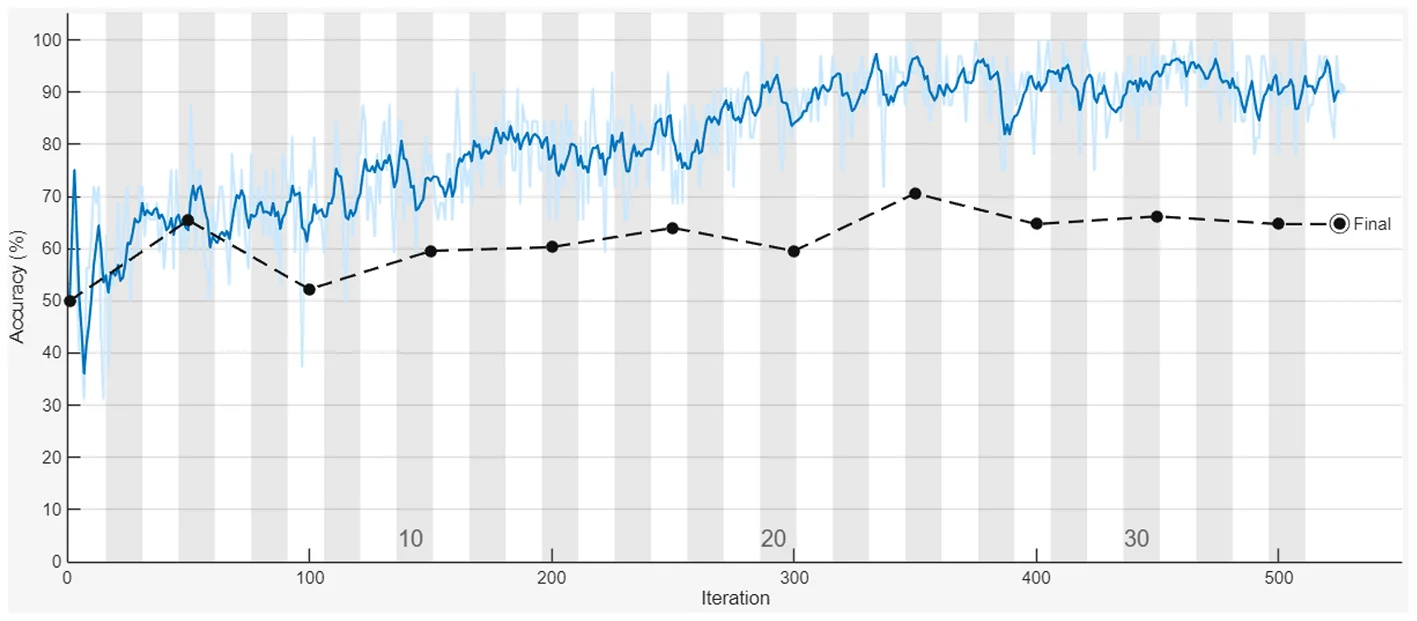
Training accuracy (Blue) and Validation accuracy (Dotted) for speaker-dependent severity detection in original dataset.

**Figure 15:**
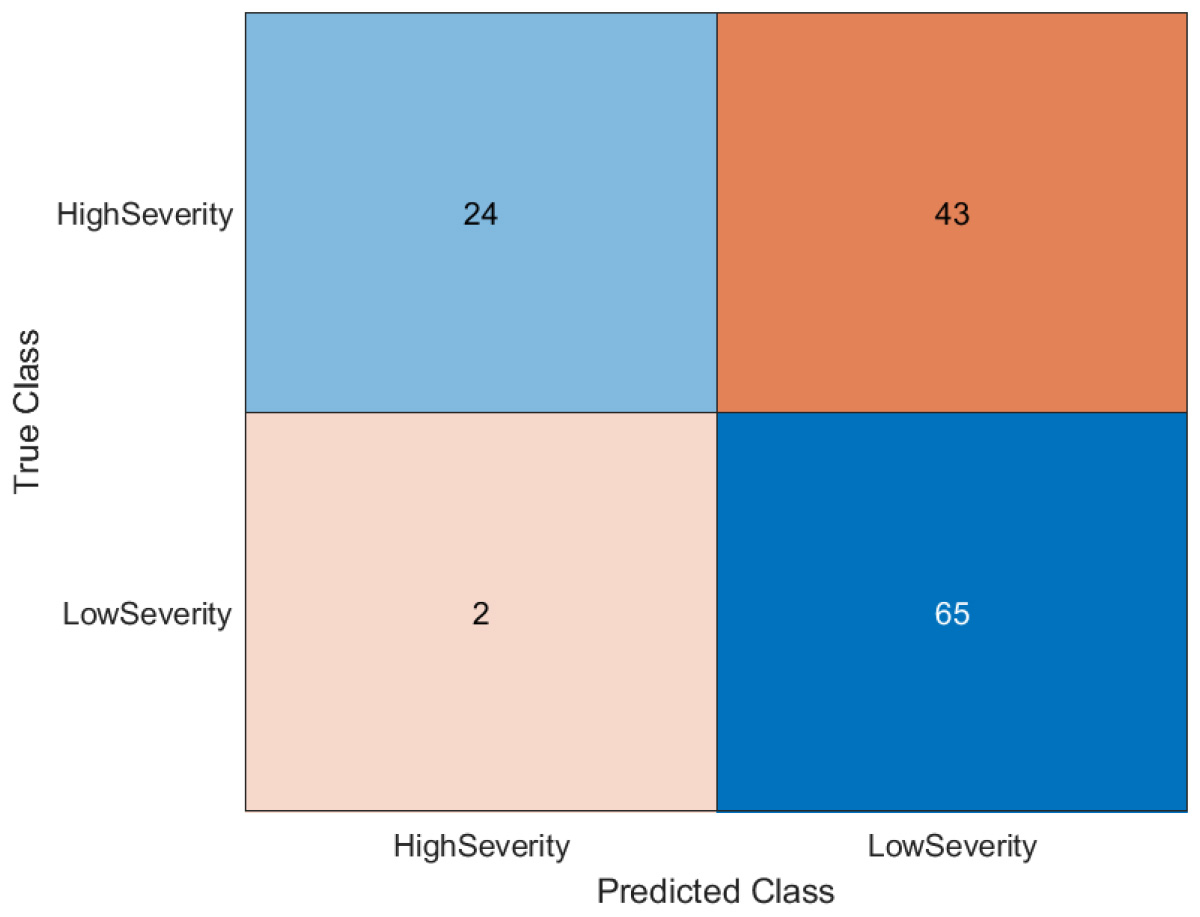
Confusion matrix for speaker-dependent severity detection in original dataset.

**Table 18:** Speaker-level performance of the original speaker-dependent low-frequency cepstrogram dataset.

Metric	Original dataset
Accuracy	86.96%
Precision	100.00%
Recall	66.67%
Specificity	100.00%
F1-score	80.00%

#### 4.2.2 GAN-generated speech vs. GAN-generated image for data imbalance mitigation

The two augmentation approaches were successful in balancing the training dataset and produced different performance profiles relative to the original imbalanced baseline, with improvements in some classification measures. GAN generated speech obtained training accuracy of 93.75%, 68.38% of validation accuracy as shown in Figure 16 and 63.43% of test accuracy. The model exhibited a precision of 64.52% and a specificity of 67.16%, while the recall was 59.70% and the F1-score was 62.02%. The confusion matrix shown in Figure 17 revealed that 40 out of 67 high-severity samples were correctly identified, while 27 high-severity samples were misclassified as low severity. This indicates a moderate ability to identify high-severity samples, with a substantial number of false-negative predictions. This implies that speech domain augmentation could generate acoustically valid samples but might not adequately maintain discriminative cepstral features needed for recognition of severe classes (Chatziagapi et al., 2019). In particular, because speaker identity is a strong and easily learned cue that is confounded with the severity label in this split, part of the observed SD accuracy may reflect speaker recognition rather than severity classification; the relatively high training accuracy of 93.75% reached by GGS as shown in Table 19 is consistent with this possibility.

**Figure 16:**
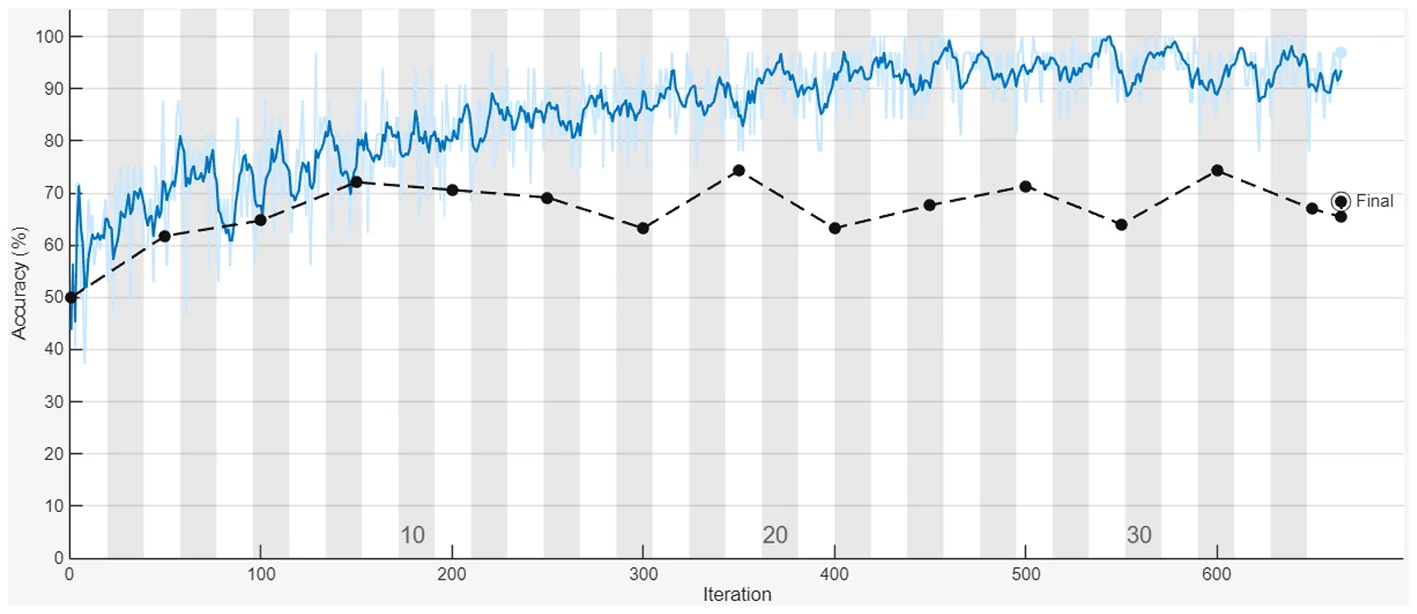
Training accuracy (Blue) and validation accuracy (Dotted) for speaker-dependent severity detection in GAN-generated speech augmentation.

**Figure 17:**
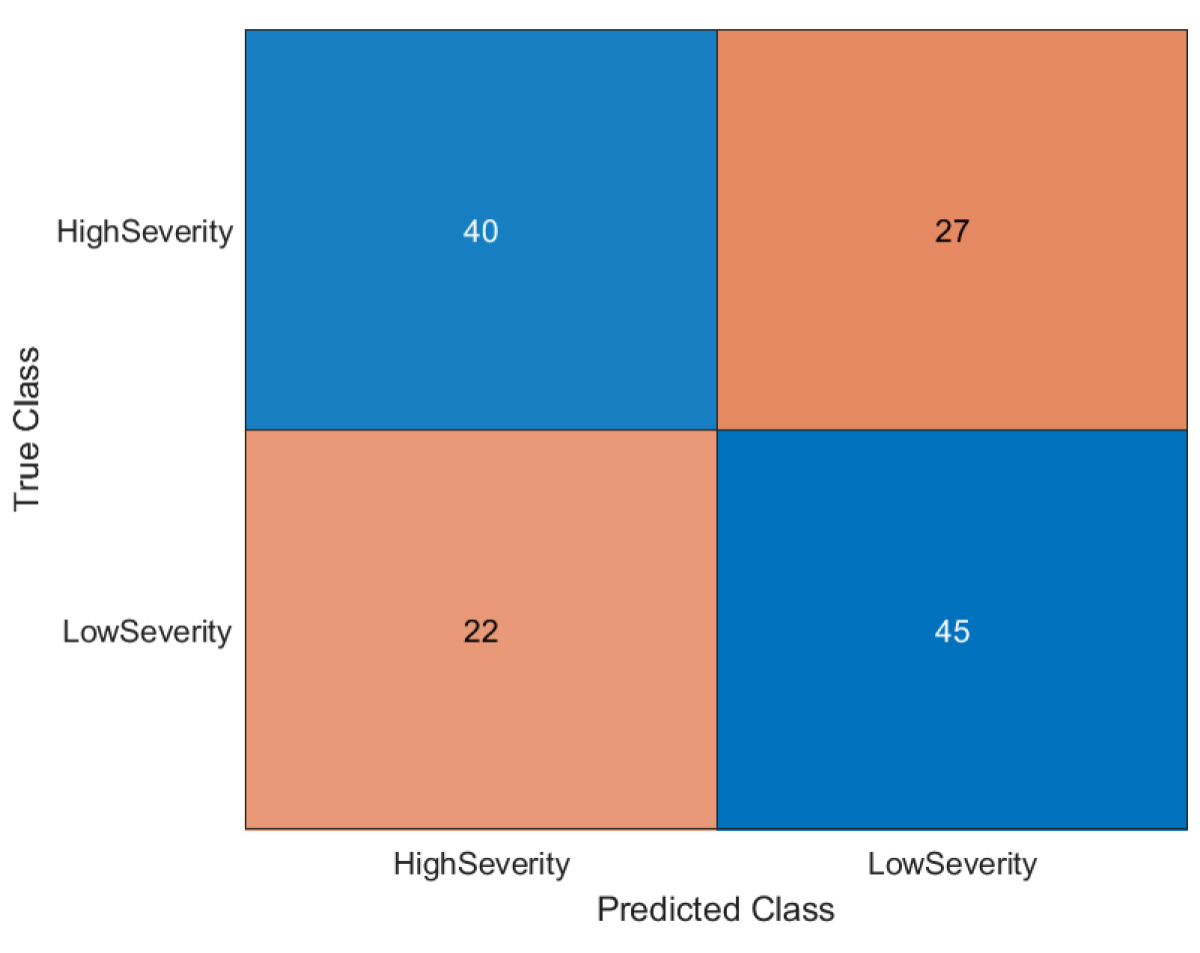
Confusion matrix for speaker-dependent severity detection in GAN-generated speech augmentation.

**Table 19:** Performance comparison of augmentation methods under speaker-dependent evaluation.

Metric	GAN-generated speech	GAN-generated image
Training accuracy	93.75%	96.87%
Validation accuracy	68.38%	75.00%
Test accuracy	63.43%	73.13%
Precision	64.52%	80.39%
Recall	59.70%	61.19%
Specificity	67.16%	85.07%
F1-score	62.02%	69.49%

The highest single-split sample-level performance was obtained using the speaker-dependent performance with the GAN-generated image augmentation, which achieved 96.87% training accuracy, 75.00% validation accuracy as shown in Figure 18 and 73.13% test accuracy, better than both GGS and the original dataset. The model achieved recall of 61.19% and F1-score of 69.49%, while maintaining balanced values for precision of 80.39% and specificity of 85.07% and the confusion matrix for the GGI augmentation is shown in Figure 19. Such balanced improvements are often desired in imbalanced clinical classification as they imply better trade-off control between missed severe cases and false alarms (Zhou et al., 2026; Huang and Jafari, 2023). The confusion matrices also validate the improved performance of GGI. GGS predicted 40 high-severity samples correctly, while GGI predicted 41 samples correctly. The false negatives were reduced slightly from 27 cases in GGS to 26 cases in GGI. In addition, GGI substantially reduced the number of false-positive predictions from 22 cases in GGS to only 10 cases. However, GGI achieved a better overall trade-off between sensitivity and specificity, leading to higher test accuracy and F1-score than GGS, despite a modest improvement in high-severity detection. This reduction in false-positive and false-negative errors indicates improved classification balance, which is especially important in clinical severity assessment applications, where both missed severe cases and false-positive classifications should be minimized (Zhou et al., 2026). As listed in Table 19, the speaker-level analysis further shows the difference in the performance of the two augmentation approaches under the speaker-dependent evaluation. At speaker level as shown in Table 20, GGS attained 78.26% accuracy, with precision, recall, specificity and F1-score values of 68.18%, 83.33%, 75.00%, and 75.00%, respectively. In contrast, GGI achieved an improved speaker-level accuracy of 82.61%, with precision, recall, specificity and F1-score values of 77.78%, 77.78%, 85.71%, and 77.78%, respectively. Thus, GGI resulted in higher overall speaker-level accuracy, precision, specificity, and F1-score than GGS, whereas GGS achieved higher recall. The higher recall of GGS indicates better identification of high-severity speakers at the speaker level, while the higher specificity of GGI indicates better identification of low-severity speakers. These findings provide an additional perspective on the sample-level evaluation and show that the relative performance of the two augmentation approaches can vary depending on the evaluation level and performance measure.

**Figure 18:**
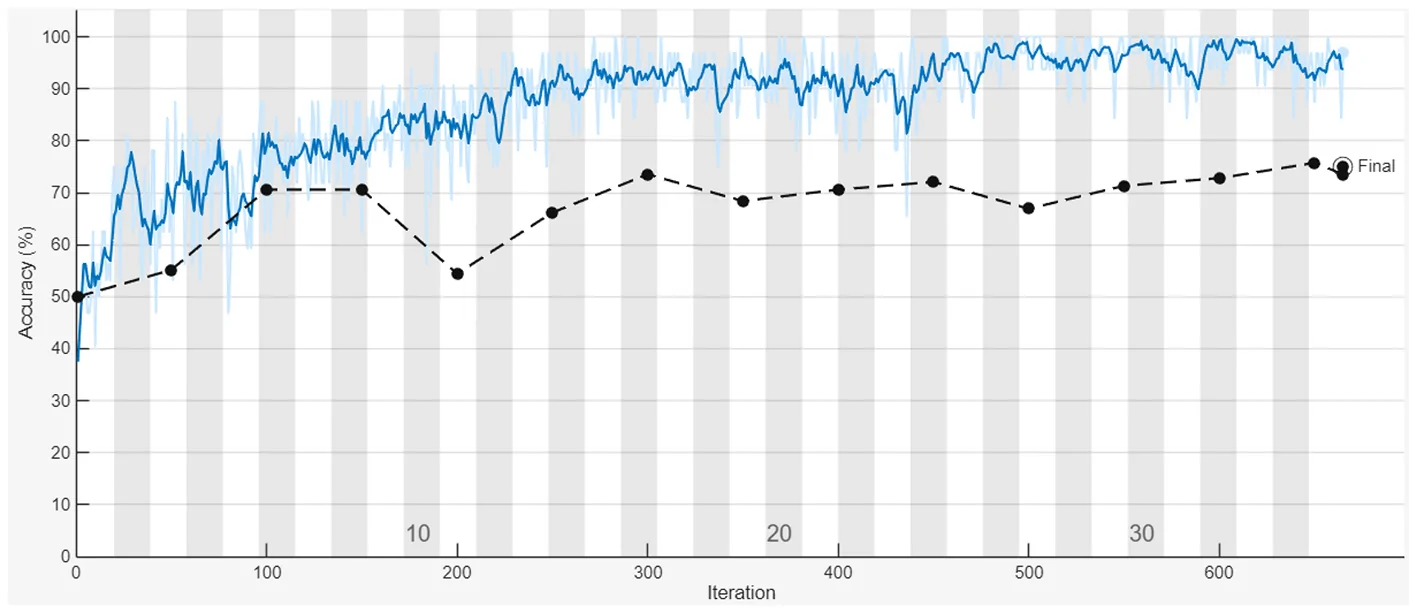
Training accuracy (Blue) and validation accuracy (Dotted) for speaker-dependent severity detection in GAN-generated image augmentation.

**Figure 19:**
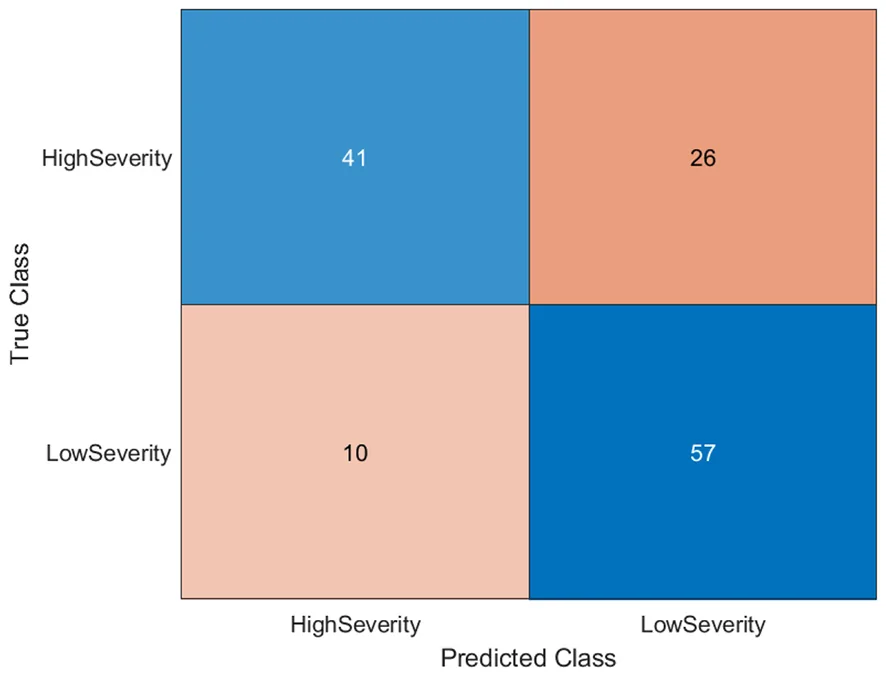
Confusion matrix for speaker-dependent severity detection in GAN-generated image augmentation.

**Table 20:** Speaker-level performance comparison of augmentation methods under speaker-dependent evaluation.

Metric	GAN-generated speech	GAN-generated image
Accuracy	78.26%	82.61%
Precision	68.18%	77.78%
Recall	83.33%	77.78%
Specificity	75.00%	85.71%
F1-score	75.00%	77.78%

To test the robustness of the speaker-dependent results, the five-fold cross-validation was repeated five times with different random seeds, thus obtaining 25 fold-seed evaluations for each experimental condition. The mean ± standard deviation across the repeated evaluations is shown in Table 21. The baseline on the original dataset achieved a mean accuracy of 73.04% ± 3.25% across the repeated five-fold evaluations. The corresponding augmented models achieved mean accuracies of 73.04% ± 3.63% for GGS and 76.48% ± 4.09% for GGI. Thus, GGS produced a mean accuracy identical to that of the baseline, whereas GGI produced a higher mean accuracy than the baseline. For GGS, augmentation increased precision and produced a small increase in specificity, while recall and F1-score were slightly reduced. For GGI, augmentation increased accuracy, precision, recall and F1-score, with specificity also increasing relative to the baseline. The standard deviations across the 25 fold-seed evaluations indicate variability across the different cross-validation partitions and random initializations. Overall, GGI showed the more favorable mean performance under the speaker-dependent evaluation protocol.

**Table 21:** Speaker-dependent performance across repeated fold-seed evaluations.

Performance measure	Baseline	GGS	GGI
Accuracy (%)	73.04 ± 3.25	73.04 ± 3.63	76.48 ± 4.09
Precision	83.04 ± 5.90	86.12 ± 4.57	85.21 ± 5.07
Recall (sensitivity)	68.00 ± 7.82	66.22 ± 8.66	71.18 ± 9.17
Specificity	77.75 ± 6.89	78.15 ± 6.20	80.68 ± 8.40
F1-score	68.08 ± 6.89	66.87 ± 7.41	72.91 ± 7.17

McNemar's exact test was used to compare the paired out-of-fold predictions of each augmented model with its corresponding original-dataset baseline, as shown in Table 22. For GGS, 266 samples were correctly classified by the baseline but incorrectly classified by GGS, whereas 266 samples were incorrectly classified by the baseline but correctly classified by GGS (*b* = 266, *c* = 266, and *p* = 1.00). Thus, there was no statistically significant difference in the paired classification decisions between the baseline and GGS. For GGI, 218 samples were correctly classified by the baseline but incorrectly classified by GGI, whereas 333 samples were incorrectly classified by the baseline but correctly classified by GGI (*b* = 218, *c* = 333, and *p* = 1.10 × 10^−6^). The GGI comparison therefore showed a statistically significant difference in paired classification decisions, with more discordant predictions favoring GGI than the baseline. Overall, the paired out-of-fold analysis indicates that GGS did not produce a statistically significant change in classification decisions relative to its corresponding baseline, whereas GGI produced a statistically significant change, with the discordant predictions favoring GGI. However, because the same fixed test samples were evaluated repeatedly across the fold-seed evaluations, pooling the predictions can treat correlated observations as independent and may result in anticonservative significance estimates. To address this dependence, paired *t*-tests were performed across the five independent seeds within each fold using the fixed real test set is shown in Table 23. For GGS, the accuracy difference from the baseline of all five folds was not statistically significant (*p* = 0.297, 0.305, 0.535, 0.345, and 0.463 for folds 1–5, respectively). In contrast, GGI yielded significantly higher accuracy than the baseline in folds 1 (*p* = 0.019), 2 (*p* = 0.038), and 5 (*p* = 0.031), whereas the differences were not statistically significant in folds 3 and 4. These results indicate that the effect of augmentation varies across speaker-stratified training partitions, with GGI showing more consistent accuracy gains than GGS under the speaker-dependent evaluation.

**Table 22:** McNemar's exact test for paired speaker-dependent predictions.

Comparison	Baseline correct/ Augmented model Incorrect (*b*)	Baseline incorrect/ Augmented model Correct (*c*)	Exact *p*-value	Significance
Original dataset vs. GGS	266	266	1.00	Not significant
Original dataset vs. GGI	218	333	1.10 × 10^−6^	Significant

**Table 23:** Per-fold paired *t*-test results for the speaker-dependent evaluation using the fixed real test set.

Fold	Original dataset vs. GGS	Original dataset vs. GGI
1	Not significant, *p* = 0.2972	**Significant**, *p* = 0.0190
2	Not significant, *p* = 0.3054	**Significant**, *p* = 0.0384
3	Not significant, *p* = 0.5347	Not significant, *p* = 0.8565
4	Not significant, *p* = 0.3446	Not significant, *p* = 0.2222
5	Not significant, *p* = 0.4627	**Significant**, *p* = 0.0310

Bold values indicate statistically significant differences between the compared methods (*p* < 0.05), based on paired *t*-tests.

### 4.3 Comparative analysis of augmentation strategies

Comparing GGS and GGI reveals that the performance of GAN-based augmentation depends on the evaluation protocol and target performance metric. In the single-split speaker-independent experiment, GGI obtained higher validation accuracy, test accuracy, precision, recall, specificity, and F1-score than GGS at the sample level. At the speaker level, both approaches achieved the same accuracy, whereas GGS achieved higher precision and specificity and GGI achieved higher recall and F1-score. Thus, the speaker-level results indicate a trade-off between the two augmentation approaches, with GGS providing more conservative predictions and GGI providing greater sensitivity to high-severity speakers. The repeated five-fold speaker-stratified evaluation with five independent random seeds, however, showed that GGS achieved the highest mean accuracy, precision and specificity, whereas GGI achieved higher mean recall and a marginally higher mean F1-score than GGS. Specifically, GGS achieved a mean accuracy of 56.47 ± 3.40%, compared with 55.47 ± 3.35% for GGI. GGS also achieved higher mean precision and specificity while GGI achieved higher mean recall and a marginally higher mean F1-score. The improvement in mean accuracy relative to the original baseline was relatively small compared with the variability across folds and random seeds. Therefore, the repeated evaluation does not suggest a consistently better augmentation strategy, but rather that GGS led to higher mean accuracy, precision and specificity, while GGI led to higher mean recall and a slightly higher F1-score under the speaker-independent protocol. The respective McNemar's exact tests showed statistically significant differences in paired classification decisions between GGS and GGI and their original-dataset baseline, with the discordant predictions favoring the respective augmented models. These findings further indicate that the two augmentation approaches altered classification behavior relative to the original imbalanced condition, but the statistical tests were not used to directly compare GGS against GGI. Similar differences between augmentation strategies have been found in speech and medical classification research, where certain methods improve recall, while others create more stable decision boundaries and better specificity (Rana et al., 2022; Zhou et al., 2026; Chatziagapi et al., 2019).

For speaker-dependent experiments, GGI again had higher validation accuracy, test accuracy, precision, recall, specificity and F1-score than GGS at the sample level. At the speaker level, GGI achieved higher accuracy, precision, specificity and F1-score, while GGS achieved higher recall. These results indicate that GGI provided a more favorable overall balance between the identification of high-severity and low-severity cases, although GGS retained an advantage in recall at the speaker level. The repeated five-fold cross-validation with five independent random seeds further showed that GGI achieved higher mean accuracy, recall, specificity, and F1-score than GGS, whereas GGS achieved higher mean precision. The corresponding McNemar's exact test showed that the difference between GGI and its original-dataset baseline was statistically significant, whereas the difference between GGS and its corresponding baseline was not statistically significant. Overall, these findings indicate that GGI provided the more favorable overall performance under the speaker-dependent evaluation protocol, although the speaker-level results demonstrate that GGS may still offer an advantage for recall. The slightly fewer false-negative predictions achieved with GGI at the sample level under the speaker-dependent protocol are consistent with the possibility that direct generation of the low-frequency cepstrogram representation may provide useful discriminative patterns for CNN-based severity classification. Previous works on image augmentation with GAN have also shown that synthetic image generation can better capture texture variation of minority classes and improve class discrimination of CNN-based systems (Rana et al., 2022; Huang and Jafari, 2023). The Grad-CAM visualizations for low-severity samples are shown in Figure 20a, while those for high-severity samples are shown in Figure 20b. These visualizations provide a qualitative indication of the regions contributing to the CNN predictions. The Grad-CAM results are presented as representative examples of the model's activation patterns and are intended to provide qualitative insight into the regions considered by the CNN during classification. In addition, a comparison with conventional class-imbalance handling and non-GAN augmentation strategies was performed using class-weighted cross-entropy (CWCE), SMOTE, and SpecAugment-style masking applied to the low-frequency cepstrogram images on the original dataset under both speaker-independent and speaker-dependent protocols, as shown in Table 24. In CWCE, the class weights were calculated from the training data only and were 1.454545 and 0.761906 for the high-severity and low-severity classes, respectively. For SpecAugment-style masking, a maximum frequency mask of 12, a maximum time mask of 16, two frequency masks, two time masks, and an augmentation probability of 0.80 were used. In the fixed speaker-independent evaluation, CWCE achieved 49.17% accuracy, 47.06% precision, 13.33% recall, 85.00% specificity, and 20.78% F1-score, whereas SMOTE obtained 50.83% accuracy, 51.85% precision, 23.33% recall, 78.33% specificity, and 32.18% F1-score. SpecAugment-style masking achieved 47.50% accuracy, 48.24% precision, 68.33% recall, 26.67% specificity, and 56.55% F1-score. Thus, SpecAugment achieved substantially higher recall and F1-score than CWCE and SMOTE, but at the cost of lower accuracy and specificity, indicating a greater tendency toward high-severity predictions. In comparison, GGS and GGI achieved 59.17% and 61.67% accuracy, respectively, in the same fixed speaker-independent evaluation, with GGI showing more favorable performance across the evaluated measures than GGS. For the fixed speaker-dependent evaluation, CWCE achieved 67.16% accuracy, 76.74% precision, 49.25% recall, 85.07% specificity, and 60.00% F1-score, whereas SMOTE achieved 65.67% accuracy, 78.38% precision, 43.28% recall, 88.06% specificity, and 55.77% F1-score. SpecAugment-style masking achieved 59.70% accuracy, 61.02% precision, 53.73% recall, 65.67% specificity, and 57.14% F1-score. It was observed that SpecAugment has higher recall than SMOTE and CWCE but lower precision, specificity and accuracy than the traditional approaches. GGS and GGI achieved 63.43% and 73.13% accuracy, respectively, in the fixed speaker-dependent evaluation. GGI therefore achieved higher accuracy than CWCE, SMOTE, and SpecAugment in this evaluation, while CWCE and SMOTE retained higher precision and specificity for some measures. Overall, the results demonstrate that the different imbalance-handling and augmentation strategies produce distinct performance profiles. SpecAugment-style masking substantially increased recall in the speaker-independent evaluation but also reduced specificity, whereas CWCE and SMOTE provided higher specificity at the expense of recall. The GAN-based pipelines achieved higher accuracy than these approaches in the fixed speaker-independent evaluation and, in the speaker-dependent evaluation, GGI achieved the highest accuracy among the evaluated methods. These results suggest that the effectiveness of an imbalance-handling or augmentation strategy depends on the evaluation protocol and the performance measure considered rather than consistently favoring a single approach.

**Figure 20:**
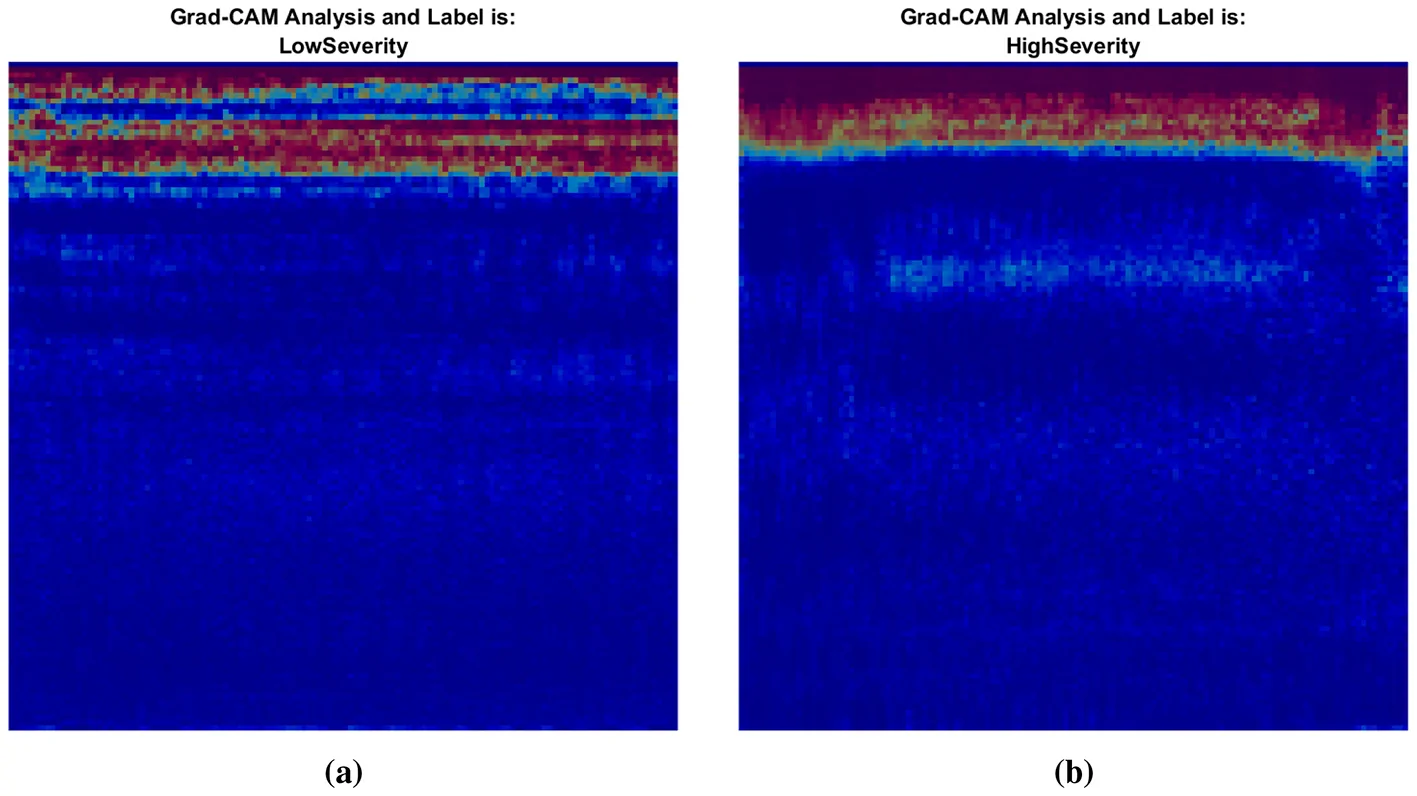
**(a)** Grad-CAM visualization for low severity. **(b)** Grad-CAM visualization for high severity.

**Table 24:** Comparison of conventional class-imbalance handling and SpecAugment-style masking under speaker-independent and speaker-dependent evaluation.

Metric	SMOTE		CWCE		SpecAugment	
	Speaker- independent	Speaker- dependent	Speaker- independent	Speaker- dependent	Speaker independent	Speaker- dependent
Accuracy (%)	50.83	65.67	49.17	67.16	47.50	59.70
Precision (%)	51.85	78.38	47.06	76.74	48.24	61.02
Recall (%)	23.33	43.28	13.33	49.25	68.33	53.73
Specificity (%)	78.33	88.06	85.00	85.07	26.67	65.67
F1-Score (%)	32.18	55.77	20.78	60.00	56.55	57.14

### 4.4 Overall findings

The main aim of this work was to find an effective augmentation technique to address class imbalance for severity classification based on low-frequency cepstrograms. The results indicate that both the GAN-generated speech and the GAN-generated image augmentation balanced the datasets and improved some of the classification metrics compared to the original imbalanced baseline. The comparative results showed that the relative performance of GGI and GGS depended on the evaluation protocol and target performance metric. In the repeated speaker-independent evaluation, GGS achieved higher mean accuracy, precision and specificity than GGI, whereas GGI achieved higher mean recall and F1-score. In the speaker-dependent evaluation, GGI showed more favorable overall performance across several classification measures, although GGS achieved higher recall in the speaker-level analysis and showed comparable performance to its baseline in the repeated evaluation. However, the speaker-dependent results should be considered as an upper-bound assessment since speaker identity is common to training and evaluation partitions. Thus, the speaker-independent results are a more suitable reference for the evaluation of generalization to unseen speakers. Overall, the results support the potential utility of GAN-based augmentation to address the problem of class imbalance, with the relative advantages of GGS and GGI depending on the evaluation protocol and performance measure in the low-frequency cepstrogram-based classification framework evaluated. Also, the performance reported for both augmentation approaches supports the potential use of GAN-based data augmentation for CNN-based classification, as demonstrated in prior studies (Rana et al., 2022; Huang and Jafari, 2023; Chatziagapi et al., 2019). To investigate the two augmentation techniques further, t-SNE was used to visualize the distribution of the feature vectors of real and GAN-generated data. Figure 21a shows substantial overlap between the GAN-generated cepstrogram images and the real cepstrogram images in the t-SNE projection, suggesting that the synthesized images exhibit feature representations similar to those of the original cepstrogram images. Whereas in Figure 21b, the spread of the distribution of the GAN-generated speech samples is observed, which indicates greater variability in the feature distribution during speech synthesis. The classification results obtained with the two augmentation approaches may be related to differences in the correspondence between real and synthetic feature representations. To further quantify the similarity between real and GAN-generated samples, Kernel Inception Distance (KID) and nearest-neighbor analyses were conducted using features extracted from the average-pooling layer of Inception-v3. As shown in Table 25, the KID for GGS was 0.197448, with a 95% bootstrap confidence interval (CI) of [0.184282, 0.214716], whereas the KID for GGI was 0.263333, with a 95% bootstrap CI of [0.249141, 0.281376]. As GGS and GGI have different representations and resolutions and Inception-v3 features do not provide a calibrated KID scale for these non-natural-image representations, the KID values were interpreted separately in each augmentation pipeline and were not used for direct quantitative comparison between GGS and GGI. The mean nearest neighbor distances were 14.3605 for GGS and 13.3092 for GGI and the minimum distances to the closest real samples were 11.1264 and 10.5920, respectively, for nearest neighbor analysis. The ratios of unique nearest neighbors were 0.1667 and 0.0800 for GGS and GGI, respectively, corresponding to 25 and 12 unique real nearest neighbors respectively. The nearest-neighbor results indicate that GGI generated samples had closer sample-level correspondence to individual real samples but that these correspondences were concentrated on a smaller subset of real samples, whereas GGS showed greater diversity in the real samples serving as nearest neighbors. These analyses provide complementary quantitative evidence regarding the relationship between real and GAN-generated samples and supplement the t-SNE visualization. Importantly, similarity to the real-data distribution does not necessarily translate into improved classification performance, as the usefulness of generated samples depends on their discriminative characteristics for severity classification.

**Figure 21:**
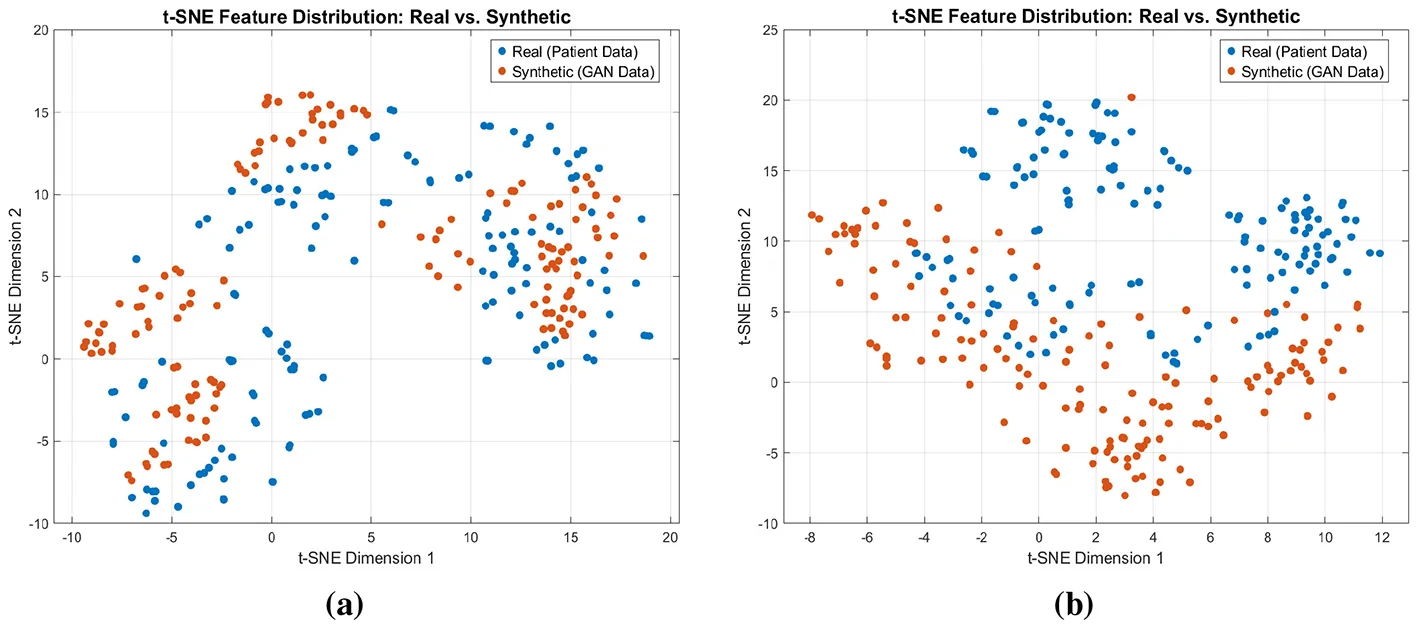
**(a)** t-SNE visualization of a GAN-generated image. **(b)** t-SNE visualization of a GAN-generated speech.

**Table 25:** Quantitative similarity measures for real and GAN-generated samples.

Measure	GGS transformed cepstrogram	GGI
KID	0.197448	0.263333
95% bootstrap CI	[0.184282, 0.214716]	[0.249141, 0.281376]
Mean nearest-neighbor distance	14.3605	13.3092
Median nearest-neighbor distance	14.4093	13.3015
Closest-pair distance	11.1264	10.5920
Unique-nearest-neighbor ratio	0.1667	0.0800

The test accuracies obtained in this study are lower than those recently reported for dysarthria severity classification on datasets such as UA-Speech and TORGO, where some studies using hybrid deep architectures have reported multi-class severity accuracies exceeding 95% (Javanmardi et al., 2024b; Varma et al., 2025; Remya et al., 2025). This difference may be related to several task-level and dataset-level factors. First, the present study uses a binary severity division based on the UPDRS speech score, with the boundary between scores of 0–1 and ≥2 occurring within a continuous, expert-rated clinical scale. This may introduce greater label ambiguity, particularly for speakers whose clinical characteristics are close to the severity boundary. In contrast, some previous studies have considered severity categories with greater separation between the defined groups. Second, continuous and read-speech tasks contain durational, rhythmic, and prosodic information, including speaking rate, pause structure, and intonation, which may provide additional information related to dysarthria severity and can be exploited by CNN- or sequence-based models (Moya-Galé and Levy, 2019; Zhang, 2025). The present study intentionally focuses on sustained vowel phonation, thereby emphasizing phonatory and spectral-envelope characteristics. This provides a more focused assessment of laryngeal-phonatory involvement, while limiting the range of complementary speech cues available for severity classification compared with continuous or read-speech tasks. Therefore, the lower absolute accuracy observed in the present study should be interpreted in the context of the specific task design, sustained-vowel input, binary severity definition, and relatively small cohort, rather than being attributed solely to the augmentation strategies. The relevant comparison for this study is therefore the relative performance of GGI and GGS against the matched original-dataset baseline, as shown in Tables 10, 17, rather than the absolute accuracies reported for different tasks, datasets, and label granularities in the broader literature.

### 4.5 Limitations

There are certain limitations in the present study. The speech-domain and image-domain GAN pipelines differ in architecture, objective function, resolution, and training configuration; therefore, the reported differences between GGS and GGI cannot be fully separated from differences in the respective generator formulations. Although the synthetic samples were assessed using distributional and nearest-neighbor analyses at the cepstrogram level, the reconstructed speech was additionally examined through an informal qualitative listening check of the basic intelligibility of the sustained vowels. This listening check was intended only for qualitative inspection and should not be considered an objective validation of the reconstructed waveform. Objective acoustic and perceptual measures, such as PESQ and STOI, were not evaluated; therefore, the perceptual and acoustic fidelity of the reconstructed GGS speech is not quantitatively assessed. Furthermore, although class-weighted cross-entropy, SMOTE, and SpecAugment-style masking were included as comparative approaches, other non-generative strategies, such as random oversampling, were not evaluated. Further comparisons with these approaches would help establish whether the observed performance characteristics of GAN-based augmentation provide advantages beyond alternative strategies for addressing class imbalance. Another limitation relates to the potential influence of utterance duration on the generated speech representations. While duration is not provided as an explicit input to the CNN and all low-frequency cepstrograms are resized to a fixed 128 × 128 representation, characteristics related to duration may still be implicitly encoded in the learned representation. A dedicated duration-controlled experiment would be necessary to fully separate duration effects from other acoustic features of the generated speech, but the additional duration-only analysis did not show that duration alone explained the training-set severity classification.

## 5 Conclusion

This study evaluates the performance of two augmentation approaches based on GAN, GAN-Generated Speech (GGS) and GAN-Generated Image (GGI) to solve the problem of class imbalance in severity detection using low-frequency cepstrograms. The original dataset is characterized by a class imbalance between high-severity and low-severity samples, that is, 300 and 450 high-severity and low-severity samples, respectively. This imbalance can negatively influence the learning process and cause a classifier bias to the majority class. To overcome this limitation, both augmentation approaches produced balanced datasets, with equal number of samples per severity class. The comparative evaluation was performed in both Speaker-Independent (SI) and Speaker-Dependent (SD) protocols to study the performance of the augmentation strategies under different speaker conditions. The results show that GAN-based augmentation improved several classification metrics in the evaluated settings although the magnitude and direction of improvement varied across augmentation methods and evaluation protocols. In the single-split speaker-independent evaluation, GGI achieved validation accuracy of 66.67%, test accuracy of 61.67%, precision of 63.46%, recall of 55.00%, specificity of 68.33% and F1-score of 58.93%. The GGS obtained 65.33% validation accuracy, 59.17% test accuracy, 60.78% precision, 51.67% recall, 66.67% specificity, and 55.86% F1-score. Thus, at the sample level, GGI showed higher observed performance across the reported classification measures than GGS in the single-split speaker-independent evaluation. Five-fold speaker-stratified cross-validation was repeated using five independent random seeds in order to assess the SI results further. Average accuracy over the repeated evaluations was 53.13 ± 3.26% for the original-dataset baseline, 56.47% ± 3.40% for GGS and 55.47% ± 3.35% for GGI. GGS had a higher mean accuracy, precision, and specificity, whereas GGI achieved higher mean recall and F1-score than GGS. The difference in mean accuracy was relatively small compared with the variability across folds and random seeds. In the single-split speaker-dependent evaluation, GGI achieved the best result, with the highest validation accuracy of 75.00%, test accuracy of 73.13%, precision of 80.39%, recall of 61.19%, specificity of 85.07%, and F1-score of 69.49%. The GGS achieved validation accuracy of 68.38%, test accuracy of 63.43%, precision of 64.52%, recall of 59.70%, specificity of 67.16%, and F1-score of 62.02%. The repeated five-fold cross-validation of speaker-dependent evaluations also showed better mean accuracy for GGI, with 76.48% ± 4.09%, compared with 73.04% ± 3.63% for GGS and 73.04 ± 3.25% for the corresponding original dataset baseline. GGI also achieved higher mean recall, specificity and F1-score than GGS, whereas GGS achieved higher mean precision. The paired statistical analysis in the speaker-dependent evaluation showed a significant difference between the original dataset and GGI, whereas the difference between the original dataset and GGS was not statistically significant. However, the same speakers contribute utterances to both the training and evaluation partitions under this protocol, and thus the SD results should be taken as an optimistic upper bound and not as a demonstration of generalization to unseen speakers. The speaker-independent results, where speakers are not shared between the training and evaluation partitions, provide a more appropriate basis for evaluating the generalization of the augmentation methods.

Hence, the results indicate that the relative performance of GGS and GGI depends on the evaluation protocol and target performance measure within the evaluated experimental framework. In the speaker-independent evaluation, at the sample level, GGI achieved higher accuracy, precision, recall, specificity, and F1-score than GGS. At the speaker level, GGS had higher precision and specificity, whereas GGI achieved higher recall and F1-score, with both approaches achieving the same accuracy. The repeated speaker-independent evaluation further showed that GGS achieved higher mean accuracy, precision, and specificity, whereas GGI achieved higher mean recall and F1-score. This demonstrates that the relative performance of the two augmentation strategies may differ across analysis levels, repeated evaluation and classification measures. Under the speaker-dependent protocol, at the sample level, GGI performed better than GGS for all reported classification measures, including accuracy, precision, recall, specificity, and F1-score. The repeated evaluations also showed higher mean accuracy, recall, specificity and F1-score for GGI than GGS, while GGS achieved higher mean precision. The corresponding paired statistical analysis showed a significant difference between the original dataset and GGI, whereas the difference between the original dataset and GGS was not statistically significant. Accordingly, these results suggest that GGI provided more favorable overall performance under the speaker-dependent protocol, whereas the repeated speaker-independent evaluation showed advantages for GGS in mean accuracy, precision, and specificity and for GGI in mean recall and F1-score. The performance differences should also be interpreted as differences between the complete augmentation pipelines, as the GGS and GGI approaches use different GAN formulations and training configurations. Therefore, GAN-based augmentation can be a potential solution to the class imbalance problem in speech severity classification. In general, the results indicate that no augmentation strategy was consistently better for all evaluation protocols and classification measures. Instead, the relative performance of GGS and GGI was dependent on the evaluation protocol, level of analysis, and classification measure. However, this comparison should be interpreted as comparing two complete GAN-based augmentation pipelines, and not as an indication that image-domain augmentation is inherently better than speech-domain augmentation. The two pipelines differ in GAN formulation, architecture, objective function, resolution, and training configuration, and these factors may contribute to the observed performance differences. Controlled experiments using the same GAN formulation and training configuration across the two domains are therefore required to isolate the independent effect of augmentation domain. Future work could explore hybrid augmentation methods combining generative approaches from the speech and image domains. The impact of the augmentation domain can be further separated from that of the GAN formulation through controlled comparisons using the same GAN architecture, objective function, and training configuration in both domains, such as WGAN-GP implemented for both speech and image generation. Additional testing of the generated speech through perceptual and objective acoustic analysis using PESQ/STOI, in combination with testing using larger speech datasets from a clinical population, may result in greater insight into the generalization capability of domain-specific GANs for speech severity analysis.

## Data Availability

The data analyzed in this study is subject to the following licenses/restrictions: The PC-GITA dataset is available upon request from Juan Rafael Orozco-Arroyave, affiliated with Universidad de Antioquia (UdeA). Requests to access these datasets should be directed to Juan Rafael Orozco-Arroyave, rafael.orozco@udea.edu.co.
